# The temporal dynamics of the Stroop effect from childhood to young and older adulthood

**DOI:** 10.1371/journal.pone.0256003

**Published:** 2023-03-30

**Authors:** Eric Ménétré, Marina Laganaro

**Affiliations:** Laboratory of NeuroPsychoLinguistic, Faculty of Psychology and Educational Sciences, University of Geneva, Switzerland; Universidad Nacional Autonoma de Mexico, MEXICO

## Abstract

The processes involved in the Stroop task/effect are thought to involve conflict detection and resolution stages. Little is known about the evolution of these two components over the lifespan. It is well admitted that children and older adults tend to show longer response latencies than young adults. The present study aims at clarifying the rational of such changes from childhood to adulthood and in aging by comparing the impacted cognitive processes across age groups. More precisely, the aim was to clarify if all processes take more time to be executed, hence implying that longer latencies rely mainly on processing speed or if an additional process lengthens the resolution of the conflict in children and/or older adults. To this aim we recorded brain electrical activity using EEG in school-age children, young and older adults while they performed a classic verbal Stroop task. The signal was decomposed in microstate brain networks, and age groups and conditions were compared. Behavioral results evolved following an inverted U-shaped curve. In children, different brain states from the ones observed in adults were highlighted, both in the conflict detection and resolution time-windows. Longer latencies in the incongruent condition were mainly attributed to an overly increased duration of the microstates involved in the conflict resolution time window. In aging, the same microstate maps were reported for both young and older adult groups. The differences in performances between groups could be explained by a disproportionally long conflict detection phase, even compressing the latest stage of response articulation. These results tend to favor a specific immaturity of the brain networks involved coupled with a slowing of the processes in children, while cognitive decline could be mostly explained by a general slowing.

## 1. Introduction

Attentional control is probably one of the most studied topics in cognitive psychology. Historically, this process has been investigated with many paradigms among which the Stroop task [1], extensively used. Even though the exact design of the task varies across studies, the Stroop paradigm can generally be defined as the presentation of two congruent or incongruent pieces of semantically related information while the subject has to “name” (manually or orally) only one of them, usually the color of the ink in which words are displayed. It is typically observed that subjects are slower to process incongruent items (e.g. naming the ink color of the word “blue” written in red) than congruent ones (e.g. naming the ink color of the word “green” written in green), and the congruent condition seems to lead to less errors than the incongruent one [2]. This difference in reaction times and accuracy is known as the *Stroop effect*. Since 1935, a large number of studies have tried to understand the core processes underlying the Stroop effect, namely how the conflictual information of the word reading and color naming processes is handled from a cognitive and neural point of view. Authors globally agree on a two levels of conflict model explaining the Stroop interference. The first one appears after the perception of an incongruency in the stimulus (stimulus conflict), while the second one is a response-based conflict, occurring when the response has to be selected among the two possible answers [3–6]. Moreover, the Stroop task involves some additional processes relative to other attentional control tasks such as the flanker or the Simon tasks. Indeed, it involves components engaged in word production as well as executive processes allowing to select the correct answer [7].

To go beyond the conclusions obtained from behavioral studies, researchers used neuroimaging approaches, such as magnetoencephalography—MEG—[8, 9] and electroencephalography—EEG—[10–21], allowing to infer on the cognitive processes and their dynamics. The results of these electro- and magnetophysiological studies tend to converge on some central conclusions. Indeed, most studies reported a more negative deflection for incongruent relative to congruent trials around 400 to 450ms associated with negative centroparietal topographies (referred to as N400 hereafter). However, some others reported the same effect later, around 500ms [21, 22], revealing the volatility of the effect. A second component has also been described for the same comparison (congruent versus incongruent trials) at a later time-period. The latter sometimes is referred to as the slow potential component (SP), late positive complex (LPC), the P600 or SP600 (SP600 hereafter), and is widely spread around 600ms, showing a centroparietal positivity. Notwithstanding converging results on the components underlying the Stroop effect, the cognitive processes behind these components have been widely debated. A general agreement has however emerged on the idea that the N400 is associated to the detection of the conflict (conflict at the stimulus level) while the second component has been associated with conflict resolution (conflict at the response level).

It is also noteworthy to mention that the vast majority of behavioral and neuroimaging studies used manual responses (mapping between the color response and a keyboard key), and very few studies had participants giving a verbal response [8, 16, 19, 23, 24]. These two response modalities do not always lead to the same results. Some studies tried to understand the electrophysiological differences characterizing the verbal and manual versions of the task. Initially, Liotti and colleagues (2000) confirmed the behavioral finding already reported in earlier studies that the Stroop effect was larger in the verbal modality [2] and showed increased N400 and SP600 effects. However, when comparing the response latencies with the timing of the components, it appears that the SP600 effect is located at the same temporality as the response time. It implies that the observed difference on the SP600 component could simply be attributable to a shift in the response artifact latencies between the congruent and incongruent conditions. Classically, to overcome this limitation, the ERP signal is aligned first to the stimulus (stimulus-aligned signal) and then to the response (starting the temporal window from the onset of the response and extracting anterior time points, i.e. response-aligned signal) [25, 26]. By using this procedure, some authors showed significant differences between congruent and incongruent conditions only for the vocal version of the task close to the production of the response [24]. These results were interpreted as an interference effect appearing not only at a semantical or lexical level, but also at the word form encoding level. In other words, the verbal and manual Stroop tasks are comparable in terms of involved cognitive processes except that the verbal Stroop task involves also a word form encoding conflict component explaining increased interference effect for this modality.

To sum up, the ERP components characterizing the cognitive processes involved in the Stroop task are globally well defined and their underlying mental processes are consistently described. However, this is only the case for young adults: other populations such as typical children and healthy older adults might show differences in mental processes and brain activations when performing the Stroop task. As further detailed below, the literature shows that the timing of the ERP components elicited by the task is modulated by the age of the participant. It is nonetheless still unclear (1) how the neurophysiological components of the Stroop effect evolving across the lifespan relate to cognitive processes underlying behavioral changes and (2) if the involved processes are identical at all ages. Hereafter we will review the available evidence on these two issues and the questions that are still debated or unresolved.

### 1.1 The Stroop effect across the lifespan

The large majority of what we know about the Stroop task comes from studies based on young adult participants. A few studies have investigated changes in executive control over the entire lifespan with the Stroop task or some other tasks [15, 27–31], but these studies are not numerous in the wide literature of the evolution of executive control abilities. That is why in the following, we will first examine the evolution of the Stroop effect from childhood to adulthood, and at a second stage, focus on the mechanisms explaining the decrease of performances in aging.

#### 1.1.1 From childhood to adulthood

The literature on the evolution of performance from childhood to adulthood suggests that even after a short exposure to reading, children show a strong and reliable Stroop effect despite longer latencies and an increased error rate [2, 32]. In school-age children, the Stroop effect evolves in a non-linear pattern, which seems entirely related to reading acquisition as when accounting for reading level, a linear trend appears [33]. This observation involves that the development of attentional control and reading abilities interact in the evolution of the performances on the Stroop task across childhood. Using an ERP approach with children aged from 6 to 12 years old performing a semantic Stroop task (e.g. naming the font of the word “sky” written in blue), Jongen and Jonkman [34] claimed that stimulus conflict (reflected by the N400 component) appears early in the development, which translates by an early setup of the inhibition mechanisms, while the response conflict (reflected by the SP600) continues to mature until 12 years old. This result is in accordance with a literature review, stressing that the processes at play when handling interference are not fully developed before the age of 12 years or later [35], as well as a study investigating the evolution of the conflict adaptation (adaptation of the attentional control system from one trial to the next) [36]. The literature on other attentional control tasks such as the flanker task suggests that conflict detection processes reach their maturation at the age of 10 years old while, surprisingly, conflict resolution does not seem to evolve. The absence of evolution of conflict resolution processes could be related to the non-verbal nature of the task [37]. Overall, authors conclude that since the ERP components are already present in childhood, processes are equivalent but differ in their maturational status. However, even though processing speed evolves during development, it cannot explain the entire changes observed between childhood and adulthood [38].

#### 1.1.2 Aging

At the other end of the lifespan, it is well admitted that cognitive performances decline, and this is particularly well described regarding conflict processing abilities. Different explanations have been proposed regarding the general alteration of cognitive performances with aging. Two hypotheses have been suggested: the general slowing hypothesis and a specific alteration of executive functions with aging. These hypotheses were derived partly from the decline observed in the Stroop task.

The general slowing hypothesis explains the longer latencies in aging by a slowdown of all processes. The decline could then simply be explained by a decrease in attentional resources but a stability of the inhibition abilities [39–43]. Applied to the Stroop task, this would imply that both stimulus and response conflicts are impacted in the same way by aging, since all implicated cognitive processes are slowed down to compensate the needed increased effort. Mathematical models of the effect of aging on the Stroop interference concluded that the general slowing hypothesis explained all the effect, leaving no room for the deterioration of a specific process [42, 43]. Likewise, more recently, a meta-analysis on several inhibition tasks, concluded that only the general slowing hypothesis explains the observed changes [40]. On top of these mathematical and statistical considerations regarding the Stroop effect’ evolution, an experimental approach manipulating the inter-stimuli delay showed that by allowing participants more time to rest, elderly subjects reach the performance of their young counterparts [39].

On the other hand, the second hypothesis claims that the main explanation for the longer latencies and increased interference effect in elderly compared to young adults is related to the decline of (a) specific cognitive process/es in aging that cannot be explained by general slowing [22, 27, 44–49]. Regarding the Stroop task, this hypothesis might imply an alteration of one or several process(es) (conflict detection or conflict resolution) more than others. This effect was shown by combining approaches of semantic Stroop task and single-colored-letter paradigms (i.e. only the central letter of the color word is displayed in color). Some authors reported a deficit in aging only in the response conflict but not in the stimulus conflict (or semantic conflict) [49]. From a neural point of view, a princeps ERP study using the Stroop task in old adults showed a modulation of the P300 component (positive component peaking around 300ms after stimulus onset and showing a centro-parietal positivity), as well as the N400, SP600 and error-related negativity components [20]. The results were interpreted as a specific decline of these mechanisms in aging, although the authors did not conclude on the origin of such decline. It seems that both stimulus and response conflict are impacted since the N400 as well as the SP600 are modulated, nevertheless further clarifications are needed to better characterize this phenomenon and to disentangle the general slowing from specific deficit theories.

At a first glance the two theories are contradictory, but it is possible that they are two faces of the same coin. All processes can be slowed down in aging due to attentional decline but some may be affected more than others, reflecting a decline in specific executive processes. Moreover, a slowdown in latencies does not exclude that compensatory mechanisms are involved. The complementarity of the two theories has been investigated using behavioral approaches [50]. Applying a hierarchical regression model, the authors observed that processing speed (measured by a simple reaction time task) accounted for a large part of the variance but age explained a specific part of the residual variance, supposing a specific deficit in executive functions, although this result may be further clarified using electrophysiological approaches.

It is worthy to mention that the majority of studies investigating the aging mechanisms of the Stroop effect mainly recruited participants over 65 years old. However, performances at the Stroop task are known to deteriorate before that age [51, 52], from 60 years old.

To sum up, state of the art literature agrees that brain activations change from childhood to aging. Indeed, components measured on the EEG signal vary across ages in amplitude and timing of the peaks. The changes in latencies and error rates in the Stroop task are generally interpreted as the consequences of less developed processes in childhood compared to adulthood and a decline in these same processes with aging. Differences in timing could translate a linear development of attentional control as well as a linear decline of the performances of the latter, but no arguments were highlighted in the neurophysiological literature regarding a specific decline of attentional control, as argued partly in the behavioral literature.

### 1.2. A single mechanism underlying congruent and incongruent trials, at least in adulthood

One of the core questions tackled in the first neuroimaging literature dedicated to the Stroop paradigm was to find how the conflict is processed and whether it requires an additional mechanism as compared to congruent or neutral trials. EEG results usually indicate similar components across conditions, except for a difference in amplitude of the N400 component and the SP600 (but see concerns about the SP600 in Stroop tasks with manual–button press–responses reported by Zahedi and colleagues (2019) and summarized above). Since latencies vary between the congruent and incongruent conditions, the amplitude difference observed between the components might be attributed to a shift in the signal rather than to different processes (i.e. differences in amplitude) underlying the two conditions. The issue of whether there is a pure shift of the same components or different brain processes across conditions is best investigated using microstates analyses. This method allows to investigate the presence of different global electric fields in the signal, i.e. of different underlying mental processes [53–56], but also their relative onset and duration. The analysis would allow to further describe whether the same processes underpin the two conditions in the Stroop task and whether this is the case across age-groups. Several studies investigated the Stroop effect using microstates [14, 57–59], but to our knowledge, only two of them have investigated the brain networks involved in both congruent and incongruent conditions. Khateb and colleagues proposed a modified Stroop task to young adult participants and segmented the ERP signal in periods of quasi-stable global electric fields [14]. Their results confirmed that the brain processes were common between congruent and incongruent trials, but that one microstate was extended in the incongruent trials compared to the other condition, reflecting differences in response latencies. The differences in latencies could thus be explained by the increased duration of a particular topography in the signal of incongruent trials. The Stroop interference would then be recruiting a large network and the results speak against a specific module at play to detect and resolve conflicts, in accordance with the rest of the literature. These results were confirmed by a second study showing the appearance of the same microstates maps for congruent and incongruent trials in the EEG signal, however showing a different distribution of the maps starting at about 500ms until 750ms. The authors performed source localization analyses on this time-window which pointed out significant activation differences located in the ACC [59]. This study hence confirmed what was reported in previous literature, namely that the ACC was sensitive to the time spent on an item rather than the presence of a conflict [7]. According to the conflict monitoring model, this structures activates in presence of conflictual information and acts as a conflict detector [60]. Nevertheless, it has been suggested that the ACC might appear more activated in the incongruent condition given its longer activation time [7, 59]. It is worth to mention that the time window carrying the Stroop effect is different in the two mentioned studies investigating the Stroop effect using microstates analyses: Khateb and colleagues showed an elongation of the microstate underlying the N400 while Ruggeri et al. reported a difference in the SP600 time window. The different loci of the effect remain unclear, but might be due to the design of the tasks, since Khateb and colleagues used a passive pseudo-Stroop task.

In the present study, we went a step further by investigating whether the same underlying processes for congruent and incongruent trials are also observed during development and in aging. As summarized above, despite behavioral and ERP changes observed across the lifespan on the Stroop task, the underlying processes seem to be the same, although less efficient during maturation and in aging. This issue has however never been confirmed directly by considering both children and older adults in a same study. More specifically, as mentioned above, microstates analysis allows to go beyond changes in the time course of the amplitude by investigating changes in topographies. Indeed topographies often tend to be more informative than waveforms analysis since they reflect brain networks including the sum of all the activated structures at a given point in time. This tool will allow to investigate if the involved brain networks are immature, if additional processes are needed to perform the task or if children simply need more time at each trial. The same questions will be raised in aging. Given the strong debate between the general slowing and specific impairment theories, the present study might help at clarifying which hypothesis is the most probable.

To address this, we designed a classical serial Stroop task including congruent, incongruent and neutral items (rows of symbols displayed in different colors) while recording the participants brain activity with high density EEG in school-age children (10 to 12 years old), young adults (20 to 30 years old) and older adults (from 58 to 70 years old). EEG signal was first analyzed with mass univariate test on the waveform amplitudes, and in a second step, using topographical or microstates analysis. Finally, source localization analysis was performed on the time-window of the microstates carrying the Stroop effect to clarify the underlying brain structure and associated cognitive processes. Even though children, young and older adults were included in the same analysis to distinguish better the different trend of development and aging, the results were interpreted separately, as distinct research questions.

According to the literature on the development of the Stroop effect reported above, we expected that children would have the same brain networks at play when performing a congruent or an incongruent trial. However, it is still unclear if children are slower and display larger Stroop effects relative to young adults because of the immature attentional resources impacting all processes or if only some processes need more time to become fully efficient. In other words, if microstates are identical in children and in young adults but are only different in duration, then it might suggest that the executive processes are already mature in school-age children but attentional resources are not developed enough to allow reaching the performances of young adults. Another possible explanation would be that one specific mechanism is still immature in children and explains the slower reaction times. This hypothesis would be confirmed if additional microstates were identified in children relative to young adults or if a specific microstate was significantly more impacted in children.

The same rationale may be applied regarding aging. A lengthening of all microstates would favor the general slowing hypothesis while either a supplementary process (additional microstate) or a different process explaining the same time window observed in older adults relative to young adults would favor respectively a compensatory mechanism or an altered one.

Given the relatively low sample size per group and the fact that the majority of the literature includes old adults rather than older adults, we performed the within-group analyses a second time on an extended group of older and old adults to ensure that the present results are not biased by age of the participants or the sample size.

## 2. Method

### 2.1 Participants

Seventy-four participants were recruited, divided into three age groups (children, young adults and older adults). Among them, only participants with a minimum of 25 artifact-free ERP epochs per condition (see pre-analyses) were retained. The final sample thus included 53 participants: 17 children from 10 to 12 years old (mean age: 11; SD = 0.79; 7 females; mean number of years of education: 7.53; SD = 1.09), 18 young adults from 20 to 30 years old (mean age: 23.9; SD = 2.97; 13 females; mean number of years of education: 16.3; SD = 2.31) and 18 older adults from 58 to 70 years old (mean age: 64.9; SD = 3.6; 14 females; mean number of years of education: 14.4; SD = 2.95). Age range for children has been narrowed down (three years for children and about ten years for adults) to reduce heterogeneity in the children group. When considering Table 2 and in particular standard deviations, children, the group with the narrowest age range, showed the most variability while the standard deviation of the other two groups were comparable. Furthermore, a linear model assessing differences in educational level showed a significant main effect of group (F(2) = 12933; p <0.001). The post-hoc decomposition of this effect showed that, unsurprisingly, children had a significantly lower educational level than young (t = -153.089; p <0.001) and older adults (t = -121.007; p <0.001), and young adults had a significantly higher education level than the older adults (t = 32.5; p <0.001).

Regarding the extended group of older adults, we included 13 additional “old adult” (over 70 year-old) participants, thus forming a group of 31 older/old participants, ranging from 58 to 81 years old (mean age: 69.4; SD = 3.56; 22 females; mean number of years of education: 14; SD = 3.16).

All participants were right handed [61] native French-speakers, and did not report any language, neurological, psychiatric or color vision impairment. All participants or their legal representative gave their written consent before the beginning of the procedure and received a financial compensation for their participation. The entire procedure was approved by the local Ethics Committee.

The participants were part of a larger group from which only the behavioral data has been analyzed in previous study [41].

### 2.2 Materials

A 180 trials, classical four colors Stroop task requiring verbal responses was used. The verbal stimuli were four color names in French (“*bleu*”; “*jaune*”; “*rouge*”; “*vert*”, respectively *blue*, *yellow*, *red* and *green*) as well as symbols displayed in different colors, were those of Fagot and colleagues (2008) [62]. The task set encompassed 60 congruent (the color font and the color words match, e.g. the word “*bleu*” written in blue), 60 incongruent (there is a discrepancy between the color font and the color word, e.g. the word “*vert*” written in red) and 60 neutral items (“++++”; “^^^^”; ““““; “****”) displayed in one of the four different colors. All verbal stimuli were presented in lower case at the center of the screen.

### 2.3 Procedure

Participants sat at approximately 80 cm of a 17 inches computer screen (refreshment rate: 50Hz). Stimuli were presented using the E-Prime software (E-studio). Oral responses were recorded by a microphone and sent to the E-Prime software to be recorded and labelled. To estimate reaction times, the onset of the production was manually retriggered offline using the CheckVocal software [63].

Participants received the instruction to name in which color the stimuli were displayed. They were also asked to produce their response orally as fast and accurately as possible. Regarding the instructions relative to the EEG recording, in order to avoid artifacts in the signal, the participants were asked to remain still and blink only after the end of the production of their responses.

The trial structure was identical for all the age groups. First, a white fixation cross was displayed on a black background for 500ms. To avoid visual responses’ contamination on the temporal window of interest due to the fixation cross, a black screen was presented for 200ms. The stimulus was then presented for 1500ms, followed by a variable interstimuli black screen for which the duration was ranging randomly from 1000 to 1200ms.

Before the beginning of the task, a training phase of 32 items including all the possible stimuli was proposed to the participant. The purpose was first to familiarize the participants with the task, but also to avoid, or at least attenuate, an eventual novelty effect on the first trials.

### 2.4. Behavioral data analysis

Statistical analyses, data wrangling and figures were performed using the R software (V.3.8) [64] with the libraries base, dplyr [65], tidyr [66], ggplot2 [67], lme4 [68], lmerTest [69], emmeans [70], NPL [71](available at: https://github.com/EricMenetre/NPL), optimx [72], car [73] and glmmTMB [74] packages. Data and code generating the figures and the analyses is available on the Yareta platform (https://doi.org/10.26037/yareta:f5w5go6tq5bsdn645wssekf46e).

A trial was excluded if the participant gave an incorrect color name, corrected himself/herself immediately after the production of an error, or did not give any response. Minor hesitations such as phonological transformation (example: “r-rouge”) were considered as correct. Regarding latencies, only correct trials were considered for the behavioral analysis of the latencies as well as for the EEG data analyses. Moreover, all response times under 200ms were excluded, as well as values outranging the threshold of 2.5 standard deviations above and under the mean of the age group. The respective rejected trial rates for congruent, incongruent and neutral condition was: 0.85%, 3.85% and 0.35%.

The data were analyzed using linear mixed models with the lmer function (lme4 package) for latencies and generalized linear mixed models fitting a binomial distribution with the glmer function (from the same package) regarding accuracy. Post-hoc analyses were estimated using the emmeans function performing a Tukey test. It is noteworthy to mention that since the model initially did not converge, the optimization method was changed for “nlminb”. For both models, post-hocs were estimated by a Tukey test targeting relevant comparisons. Moreover, to obtain main effects on generalized linear mixed models, the Anova function from the car package was used instead of the classical anova function from base R used for lmer models.

### 2.5. EEG acquisition and pre-analyses

The electroencephalogram (EEG) was recorded continuously during the task by 128 electrodes placed on a nylon cap following the 10–5 system. The signal was acquired by a Biosemi amplifier including two active electrodes (ActiveTwo system, Biosemi V.O.F. Amsterdam, Netherlands) at a sampling rate of 512Hz including an online DC filter ranging from 0.01 to 104Hz and a 3dB/octave slope. This system includes an active electrode used as recording reference and located in the centro-posterior region.

Regarding data cleaning, all the manipulations were performed with the Cartool software (V. 3.91) [75] and with the R software (V.3.8) [64]. The signal was first filtered by using a notch filter at 50Hz and a zero-phase shift order 2 bidirectional butterworth bandpass filter including a highpass filter at 0.3Hz and a lowpass filter at 30Hz. The signal was then downsampled at 256Hz to reduce the computing time and the number of multiple comparisons of the statistical analyses. The signal was re-referenced to average for ERP analyses. A first interpolation was performed on the entire signal for noisy electrodes following a 3D spline method [76]. The mean number of interpolated electrodes was 4.79 (SD = 1.84) (with at maximum 10 interpolated electrodes, or 7.81% of the 128 electrodes). Regarding the event related potentials (ERP) analyses, two time windows were selected: a stimulus-aligned period and a response-aligned period. The rationale of two alignment points has been presented in the Introduction [25, 26]. The data aligned to the stimulus for a shorter time period than the behavioral latencies allow to track the N400 effect; the data aligned to the response onset (backwards) allows the investigation of later stages of word form encoding. Finally, this approach is also adopted to avoid the presence of artifacts due to articulation in the EEG signal. The first period included the first 25 time frames (~100ms) before the stimulus presentation, which was taken as baseline, and the 130 time frames (~520ms) following the stimulus presentation. This stimulus-locked ERPs lasting ~520ms ensured the entire signal to be at least 100ms shorter than the fastest RTs (629ms in the C condition in young adults, see Table 1). The second window was selected backward from the response to the stimulus and lasted 100 time frames (~400ms). For the purpose of the EEG analyses, the response latencies used as onset of the response-aligned period were reduced by 100ms to avoid measuring the premises of articulation [26]. Since no automatic algorithm of artifacts correction were used, all the epochs were visually inspected and only the artifact-free epochs were retained for the averaging, and grouped according to the Stroop conditions (congruent, incongruent and neutral). Finally, a second interpolation was performed on the averaged data of each subject (mean number of interpolated electrodes: 3.38 (SD = 2.75); maximum 9 electrodes or 7.03% of the total number of electrodes) using the same method as described above but avoiding interpolation of previously interpolated electrodes or their direct neighbors. When combining the two interpolations, on average, 8.17 (SD = 3.3) electrodes were interpolated per participant and the maximum of interpolated electrodes was 16, or 12.5% of the total number of electrodes. On average, 17.48% (SD = 10.87), 26.82% (SD = 13.94) and 19.78% (SD = 13.08) of the trials were rejected for the congruent, incongruent, and neutral conditions, respectively, including the ones already rejected during the behavioral analyses. To avoid differences in signal-to-noise ratio due to imbalanced number of epochs among conditions, for each subject, the condition showing the lowest number of epochs was identified and a random selection of epochs was performed to match the number of epochs from the condition with the lowest number of valid epochs.

**Table 1:** Descriptive statistics regarding the mean latencies with their relative standard deviations, the range between the minimum and maximum reaction times observed and accuracy with its standard error.

Age groups	Conditions	Mean (SD)	Range RT	Accuracy (SE)
Children	C	813.9 (206.7)	281.7–1421.3	96.5% (0.8)
	I	962.7 (186.2)	320.6–1379.1	81.9% (2.3)
	N	793.6 (187.7)	369–1402.5	96.6% (0.9)
Young adults	C	657.1 (153)	288.3–1306.4	99.3% (0.3)
	I	766.7 (157.7)	406.9–1350	93.6% (1.1)
	N	628.9 (126)	339.4–1178.8	98.7% (0.5)
Older adults	C	762.1 (153.6)	425.6–1303.4	99.6% (0.2)
	I	898.4 (155.8)	488.3–1319.7	96.8% (0.9)
	N	711.1 (123.5)	402.7–1215	100% (0)

### 2.6 EEG analyses

To ensure comparison with the rest of the literature, first, the waveforms of the signal was analyzed. This was achieved by a mass-univariate statistic. In a second step, the topography of the data was considered. It was verified that the signal was topographically consistent using a topographical consistency test before running an analysis of variance between the three conditions and age groups on the signal (tANOVA). This analysis will guide the interpretation of the microstates and will allow to relate topographical differences with those observed on the waveform analyses. The signal was then segmented in microstates and the characteristics of the microstates (presence, duration and onset) were extracted. Since these microstates tend to be difficult to relate to cognitive processes, source localization analyses were performed to identify the generators behind the most relevant brain networks identified by the microstates analyses.

#### 2.6.1. Waveforms statistics

Mass univariate tests on amplitudes were performed using the STEN software [77]. The data were analyzed using a 3X3 (three conditions, i.e. congruent, incongruent and neutral, by three age groups, i.e. children, young adults and older adults) parametric ANOVA. A spatial and a temporal criteria were applied: only periods of significance of at least 4 time frames (~16ms) on at least 4 electrodes were included. A significance threshold of 0.01 was defined and, since one test per time point was performed, a correction for multiple comparisons was applied using a Bonferroni approach.

#### 2.6.2 Topographic analysis of variance (tANOVA)

Topographic analyses were performed on both (stimulus-aligned and response-aligned) ERP signals across groups using the Ragu software [55, 56]. For all the analyses on this software, the data were normalized by the GFP, the number of randomization was set to 5000 (default value) and the significance threshold to 0.05 (default value). A test evaluating the consistency of the topographies of the averaged epochs was also performed, prior to the interpretation of the tANOVA (topographic ANOVA) results. This test aims at assessing if the signal shows consistent topographies, guarantying the reliability of the topographical analyses such as the tANOVA and the microstates analyses. First, a tANOVA was calculated including the three conditions (3X3 design: three conditions, i.e. congruent, incongruent and neutral, by three age groups, i.e. children, young adults and older adults) and then to investigate the Stroop effect, the contrast between congruent and incongruent conditions was calculated in a separate analysis (2X3: 2 conditions by three age groups).

#### 2.6.3. Microstates analysis

The microstates analysis allows to decompose the EEG/ERP signal in periods of topographical stability (or quasi-stability). It has been proposed that when a cognitive process is taking place, the topography of the scalp EEG remains stable, reflecting the activation of a specific brain network. During this stability, different brain areas are connected according to one or several firing frequency/ies [78–81]. Each of these periods, or microstates, can be segmented from the evoked potentials and are known to reflect the activation of a network, generally underlying one or several cognitive processes.

Microstates analysis was performed separately on stimulus aligned and response aligned ERP signals across groups using the Ragu software [55, 56]. The software performed a segmentation on the averaged ERP of each subject using a cross-validation method based on the topographical atomize and agglomerate hierarchical clustering (TAAHC) algorithm, for a pre-defined by the user range of topographies (here from one to 30). At each fold (data are segmented in subsamples named folds and models are trained on a majority of the folds and tested on the rest), the segmentation is operated on the training data and the global explained variance is reported on both training and test data. The user then choses the more parsimonious number of template maps explaining the most variance in the signal, usually on the test data to avoid overfitting. For display, the selected maps, after applying a smoothing method (aiming at avoiding small microstates appearing in the middle of another larger one), are fitted on the grand average for each condition and age group. For a more detailed explanation of the procedure, see: [55, 82]. It is noteworthy to mention that to avoid fitting microstates related to the P1 later in the signal, the segmentation on the stimulus-aligned data was performed only after the P1 window (73 time frames, or ~192ms). This timing was based on the peak of dissimilarity observed in the children’s grand average (since children presented the wider P1).

To extract quantifiers of the microstates such as presence/absence or duration in terms of number of time frame, given that Ragu does not allow to set a criterion of minimum duration to remove short microstate maps which are not possibly related to a cognitive process, a backfitting procedure on both the grand averages (Fig 4) and in the averages per subjects was performed on the Cartool software. A minimal duration of 5 time frames (~20ms) as well as a segment temporal smoothing of 5 time frames as well was applied. Here, only template map presence/absence, onset time and duration were considered for statistical analyses. Regarding the presence/absence, the data were analyzed by generalized linear mixed models (one for the stimulus and another for the response-aligned signal) fitting a binomial distribution. Onset was analyzed by a generalized linear mixed model fitting a gamma distribution using a log link function. Since the duration data followed a Poisson distribution (count of the number of time frames occupied by a microstate in the signal), with an over-representation of the zero value, translating the presence or absence of the topography in the subject, the use of a generalized linear mixed model fitting a zero-truncated Poisson distribution (Hurdle models) was justified. The analyses were performed using the glmmTMB function. For all models, the post-hocs have been performed using Tukey tests from the emmeans package. For models which failed to converge (all of them except for the Hurdle model on microstates duration), the optimizer function has been adapted using the “bobyqa” algorithm.

The microstate maps obtained when including all the groups were fitted on the signal of the extended group of older/old adults.

#### 2.6.4 Source localization analysis

Source localization analysis was performed using the Cartool software. MRI from the MNI-152 symmetric atlas for adults (applied to both adults groups) and a pediatric atlas (sample aged from 10 to 14 years) [83] for the children group were used. The entire brain was extracted from the MRI (combining gray and white matter) and electrodes position was coregistered on the MRI head of adults and children templates. The solution space was estimated using 6000 solution points and the lead field was calculated using a LSMAC 4-shell isotropic method. To be noted that the lead field was calculated once per age group since the calculation method takes into account the average age of the group to estimate skull thickness. To select the best inverse solution method, an estimation of the sources underlying the P100 was performed with several inverse models (minimum norm, weighted minimum norm, sDale, sLORETA, LORETA, LAURA, eLORETA), and the one providing the closest activation around the occipital areas was selected. LORETA [84], gave the closest estimation, displaying a bilateral occipital activation without engagement of the cerebellum. Inverse solutions were estimated using an inverse matrix per age group based on the grand average of each condition in the stimulus- and response-aligned signal and before computing, a spatial filter was applied. The estimation of the sources was performed based on the microstates analysis. For each of the maps of interest, the onset and offset of the map per condition was selected and the corresponding sources from the grand averages per conditions and age groups were calculated. Only the maps highlighting a significant difference in terms of presence or duration among conditions or age groups explaining the Stroop effect were retained.

## 3. Results

### 3.1 Behavioral results

**Fig 1:**
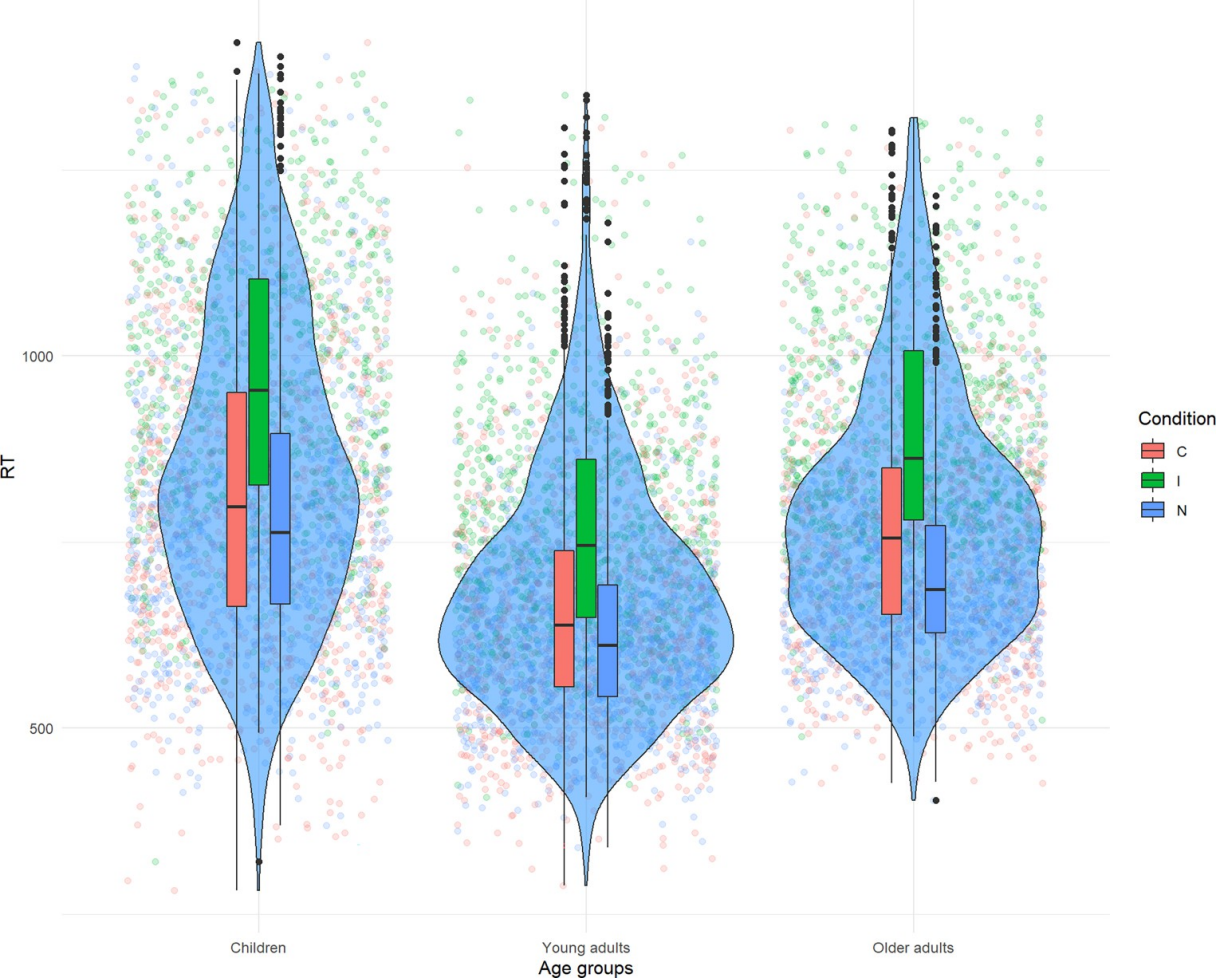
Distribution of the latencies per conditions and age groups.

As shown in Table 1, children presented the slowest latencies in all conditions (mean = 851ms; SD = 207), and young adults (mean = 682ms; SD = 157) were faster than the older adults’ group (mean = 788ms; SD = 164). Children also displayed the highest standard deviations regarding errors and reaction times, while young and older adults showed comparable variances despite differences in reaction times, as highlighted by Fig 1.

As a reminder, the linear mixed model included the conditions and the age groups as fixed effects, and the subjects ID variable as random intercept. The model highlights a significant main effect of condition on reaction times (F(2,18.9) = 111.68; *p* < 0.001), a significant main effect of age groups (F(2,50) = 10.74; *p* < 0.001), and a significant interaction between the two (F(4, 8900.7) = 13.56; *p* < 0.001). The decomposition of the interaction reveals that children (t(132.6) = -8.83; p<0.001), young (t(125.27) = -6.04; p <0.001) and older adults (t(131.73) = -8.54; p<0.001) show significantly increased latencies in the incongruent condition. As emphasized by the estimates of the model, the results show a modulation of the effect’s magnitude among conditions and age groups, suggesting that the interference effect is the strongest for children (estimate = 104.96), while it is the weakest among the young adults group (estimate = 35.98), and intermediate for older adults (estimate = 94.86). Decomposition of the main effect of groups revealed that children (t(50) = 4.58; p<0.001) and older adults (t(50) = -2.88; p = 0.016) had significantly lower latencies than young adults. Moreover, the group of children and older adults do not significantly differ (t(50) = 1.74; p = 0.201).

Regarding accuracy, the results highlight a significant main effect of conditions (χ^2^(2) = 162.44; *p*<0.001), a significant main effect of age groups (χ^2^(2) = 50.43; *p*<0.001) but no interaction between the two (χ^2^(4) = 4.8; *p* = 0.3). The decomposition of the main effect of conditions suggests that congruent trials were less prone to errors than incongruent trials (Z = 6.68; p<0.001), but none of the other comparisons reached significance. The age groups effect shows that accuracy was significantly lower in children compared to young adults (Z = -4.52; p < 0.001), however no significant differences were observed between children and older adults (Z = -0.02; p ≈ 1) nor between young and older adults (Z = -0.02; p ≈ 1).

### 3.2. ERP results

*Fig 2*.

**Fig 2:**
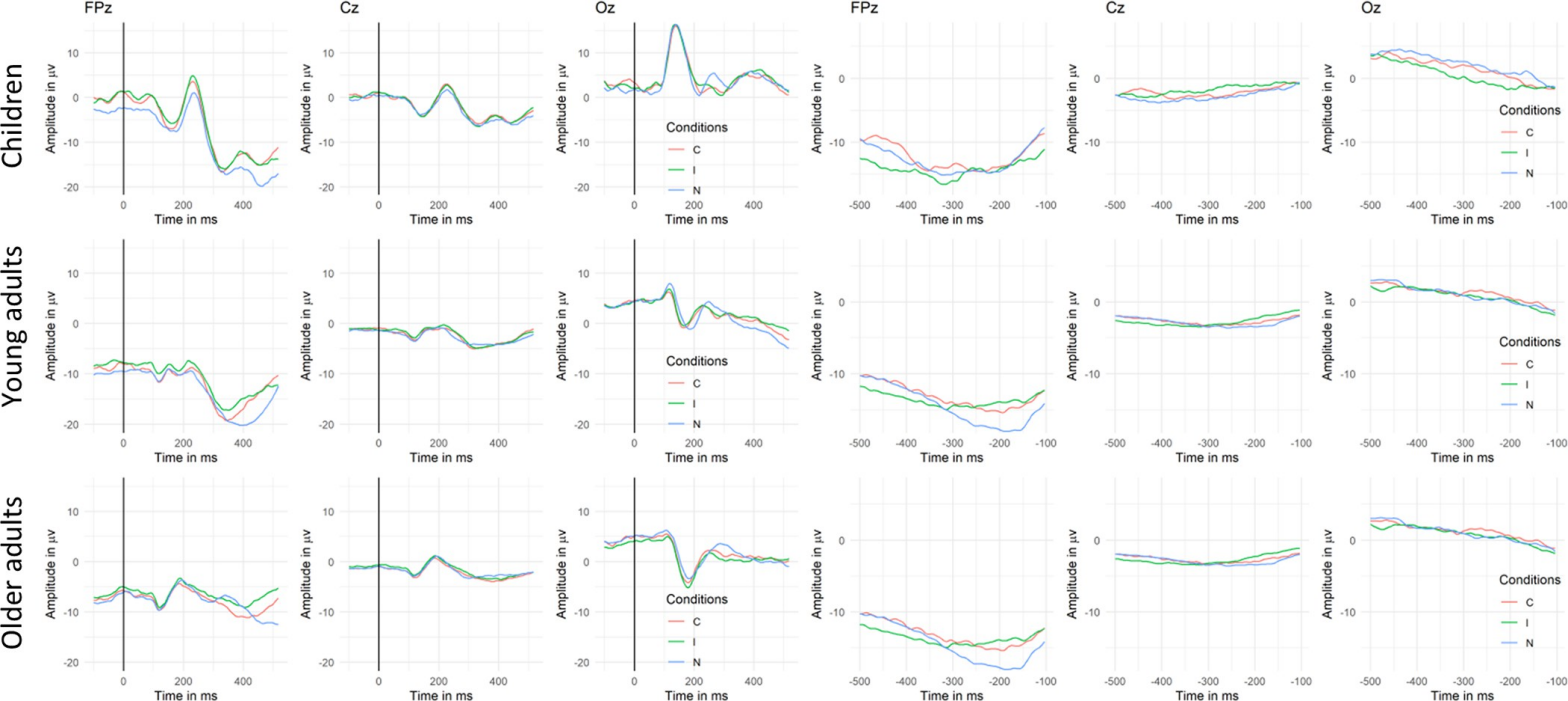
Exemplar waveforms of the grand averages of stimulus- and response-aligned ERP signal for the three conditions (C = congruent; I = incongruent; N = neutral) in each age group for the FPz, Cz and Oz electrodes. The peak analysis was also performed on the Fz and Pz electrodes. The gray rectangles shows the time window considered in the peak analysis (300 – 420ms).

#### 3.2.1. Mass univariate test and peak analysis

**Fig 3:**
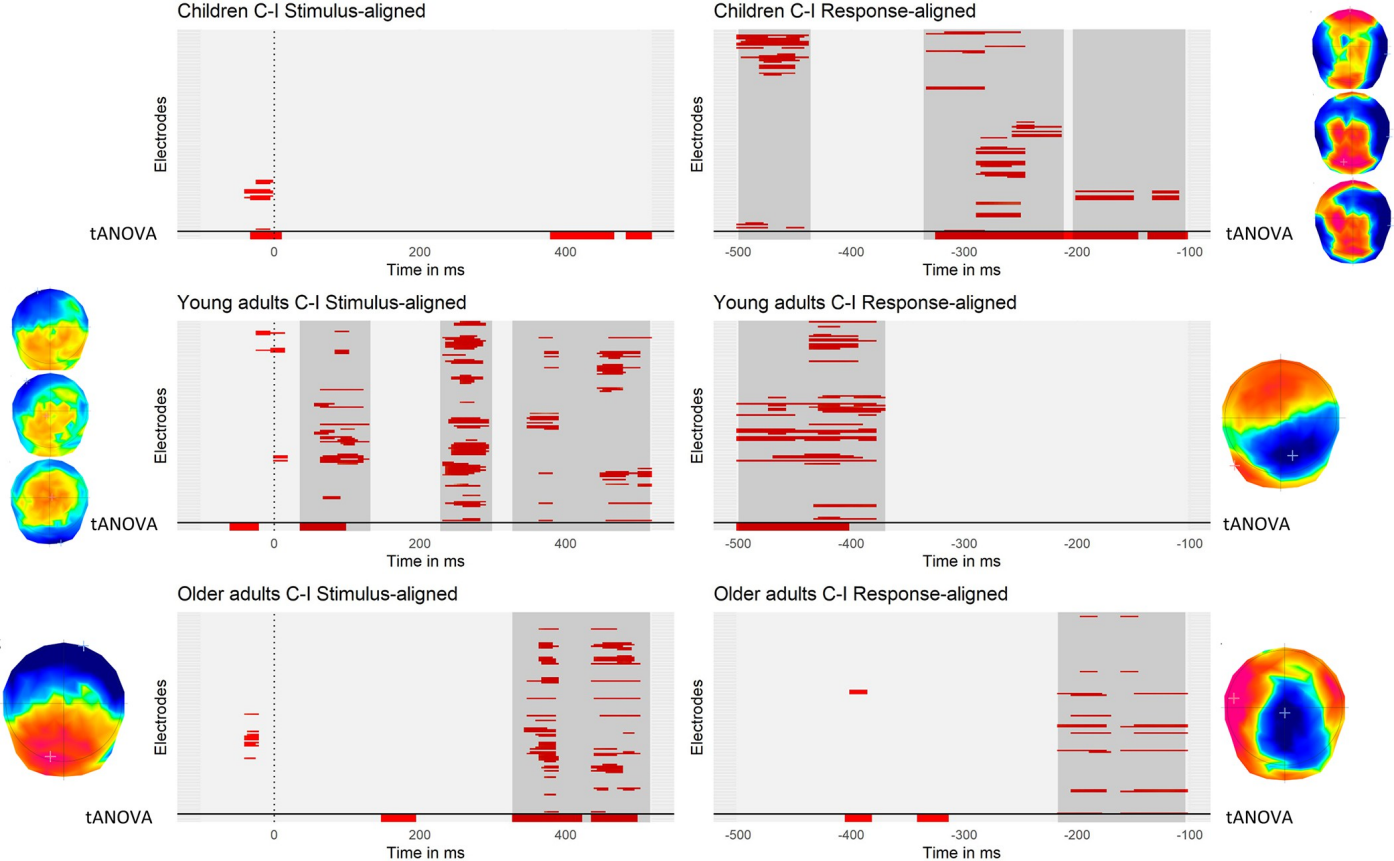
Significant differences in the contrast between congruent and incongruent conditions on each electrode and time-point for stimulus- and response-aligned ERPs within each age-group. The dotted vertical line represents the onset of the stimulus and the signal under the horizontal solid line at the bottom of each plot represents the tANOVA results specific to each contrast. Periods of consecutive significance shorter than 4 time frames (~16ms) were discarded. Clusters of significance are highlighted in gray. The topographies on the side represent the spatial distribution of the significant electrodes constituting the different clusters.

The results of the mass univariate tests on the waveform amplitudes run separately on the stimulus- and response-aligned ERPs are presented in Fig 3. Since neutral trials were very different from congruent and incongruent trials, it was suspected that most of the effect would be carried by the neutral condition compared to the other two conditions. That is what was observed since significant difference in amplitudes appeared on virtually the entire time window for both main effects of conditions and age groups as well as for the interaction as presented in S1 Fig. Since the present study focused mainly on the Stroop effect, contrasts including neutral conditions were discarded, and in Fig 3, only the contrasts between the incongruent and congruent condition are displayed for each age group.

The results of the mass univariate statistic highlighted different time windows of significant variations in amplitudes between congruent and incongruent trials in the three age groups (displayed in gray on Fig 3).

Regarding the stimulus-aligned results, the young adults group show three periods of significant difference. A first one between 35 and 132ms, a second one from around 230 to 300ms, and a third one ranging from 330 to 520ms. The latter time window was the only one also highlighted as significant in older adults. Children did not show any significant period of significant difference between the congruent and incongruent condition. Topographies of difference between the two conditions highlight a positive centro-posterior difference.

Concerning the response aligned results, children show a first period of significant difference ranging from -500ms to -436ms, a second period ranging from -336 to -212ms and a third one ranging from -200 to -100ms. Comparatively to the other groups, the first time window observed in children was also found in young adults but with a wider range (-500ms to -370ms) and different topographies of difference (antero-posterior positivity for children vs. centro-posterior right negativity for young adults). Concerning older adults, as observed for the children group, they display a period of significance close to the articulation onset (ranging from -217 to -100ms) and reversed topographies of difference (respectively positive and negative centro-posterior difference for children and older adults).

#### 3.2.2 Topographic analyses of variance (tANOVA)

The results of the topographic analysis of variance (tANOVA) displayed in Fig 3 showed different patterns across groups but were partially concordant with the mass univariate test. Regarding the stimulus-aligned signal, in children, a small time window appeared significant during the baseline, close to stimulus onset as well as about 400ms. In young adults, two small time periods suggested a change in topography, occurring in the baseline and before the P100 component, matching the mass univariate results. In older adults, several periods of significance appeared around 400ms post-stimulus. In the response aligned signal, the results of the tANOVA match the one of the mass univariate test for children showing a wide significance period ranging from -336 to -100ms while no significant topographical difference was observed for the more remote time window of waveform’s significant difference for children. The same as observed for the older adults group, for which no significant difference in topography was highlighted despite significant differences in the waveforms. Regarding young adults, both tANOVA and mass univariate results point out a significant effect ranging from -500 to -370ms.

On the extended group of older/old adults, the tANOVA showed a time window of significant differences between 284ms post stimulus to the end of the time window (520ms), in accordance to the analysis performed on the group of 18 older adults. On the response-aligned signal, Regarding the response-aligned signal, the small differences observed in Fig 3 became clearer with a significant time window ranging from -424ms to -348ms.

#### 3.2.3. Microstates analyses on the presence, duration and onset of the microstate maps

The TAAHC algorithm identified six microstates best explaining the signal on the stimulus-aligned (Maps 1 to 6) and four microstates for the response-aligned signal (Maps 7 to 10). Descriptive statistics about duration of the maps can be found in Table 2.

**Table 2:** Mean and standard deviation of the duration of each map across conditions and age groups. See S3 Fig for graphical representation.

Microstate maps	Conditions	Children	Young adults	Older adults	
Map 1	C	67.33 (61.16)	101.71 (76.69)	130.8 (91.6)	Stimulus-aligned signal
	I	73.33 (20.13)	115.33 (80.5)	185.45 (84.44)	
	N	76 (52.31)	99.33 (26.94)	165.78 (89.04)	
Map 2	C	96 (52.38)	85.6 (45.48)	130.4 (32.45)	
	I	92 (40.74)	96 (64.33)	92.8 (58.95)	
	N	116 (79.86)	61.33 (22.86)	66 (29.66)	
Map 3	C	107.43 (57.04)	115.38 (92.13)	94.4 (77.31)	
	I	106.29 (33.64)	110.77 (91.7)	103.43 (81.05)	
	N	92 (49.15)	132 (84.29)	102.67 (89.99)	
Map 4	C	122.46 (61.57)	96.67 (55.91)	113.2 (70.07)	
	I	140.67 (47.6)	130.67 (77.51)	118 (98.07)	
	N	187.2 (44.73)	83.43 (33.58)	126 (80.34)	
Map 5	C	49.6 (22.38)	138.29 (86.94)	163 (85.76)	
	I	38 (8.49)	109.54 (88.1)	188.8 (99.49)	
	N	67.2 (63.71)	147.2 (86.21)	116 (74.21)	
Map 6	C	64.8 (25.77)	102.5 (55.83)	54.67 (32.58)	
	I	70 (30.01)	86 (51.61)	136 (NA)	
	N	122.67 (52.86)	85.33 (44.94)	68 (55.57)	
Map 7	C	111.2 (23.73)	221.5 (120.78)	210.29 (100.46)	Response-aligned signal
	I	119 (62.6)	216.8 (116.32)	263.38 (94.42)	
	N	137.6 (96.57)	225.5 (121.25)	200.31 (130.99)	
Map 8	C	206.86 (106.17)	188.5 (109.07)	149.5 (86.82)	
	I	139 (86.5)	180.67 (99.64)	163.2 (65.57)	
	N	257.71 (109.54)	156.5 (106.79)	100.5 (70.05)	
Map 9	C	190.55 (102.64)	104 (107.48)	80.57 (70.93)	
	I	205.5 (95.68)	68 (55.04)	147 (95.58)	
	N	117 (94.63)	98.5 (62.59)	135.2 (74.59)	
Map 10	C	139.11 (115.29)	149.23 (128.31)	192 (111.92)	
	I	136.8 (114.83)	185.14 (104.34)	158.13 (112.28)	
	N	110 (116.27)	129.33 (136.65)	239.69 (146.49)	

The analyses performed on the microstate maps aim at extending the results of the tANOVA. Indeed, this analysis highlighted several periods of significance but it remains unclear if these differences are related to changes in topography (different microstate maps for the same time window) or if only a difference in duration or onset can explain the significance. Microstates analysis was also able to investigate expected period of significance using another approach (based on the hypotheses or differences in other age groups), which were not supported by the tANOVA. Detailed results of the generalized linear mixed models performed on the presence, duration and onset of the microstate maps can be found respectively in S1–S12 Tables. Only the relevant comparisons are reported in this section. The dynamic of the topographies is depicted in S5 Fig and dynamic of the topographies in S6 Table.

**Fig 4:**
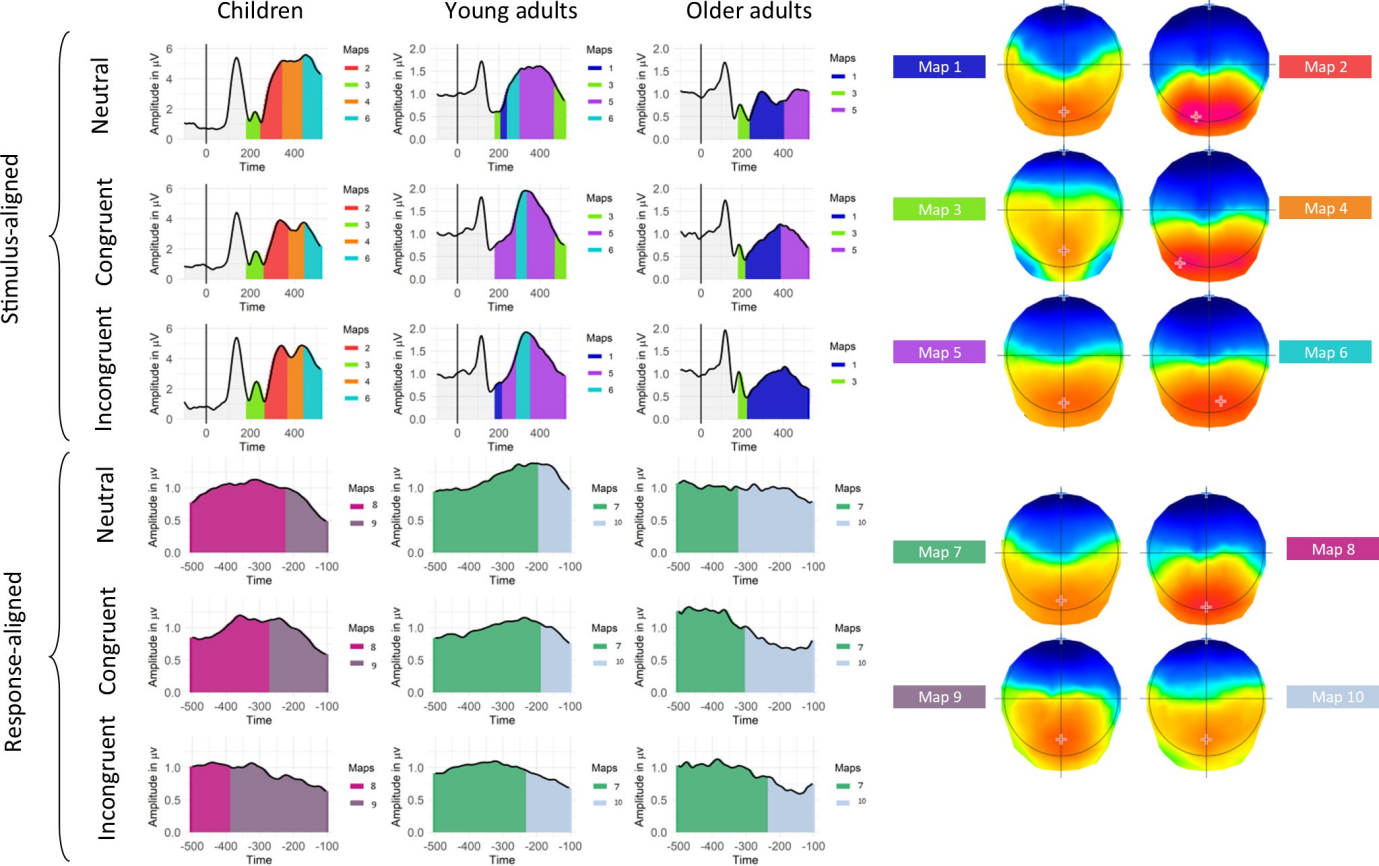
Periods of stability of the electric field at the scalp are represented under the GFP curve of the grand average ERPs with color codes for stimulus-and-response-aligned signal, for each age group and condition. The corresponding template maps are displayed on a continuum from red (positive) to blue codes (negative).

On the stimulus aligned signal, as presented in Fig 4, some of the maps tended to be specific to children, such as Map 2, present at about 300ms post-stimulus onset (children–older adults: z = 4.98, p < 0.001; children–young adults: z = 2.23, p<0.001; older adults–young adults: z = -0.19; p = 0.91). It also appeared that Map 5 was specific to the young adults group (ranging from ~200 to 520ms) (children–older adults: z = -2.02, p = 0.106; children–young adults: z = -5.17, p<0.001; older adults–young adults: z = -3.59; p<0.001). Another microstate map (Map 6: 450-520ms in children and 290-350ms in young adults), was found significantly more often in the signal of children and young adults (children–older adults: z = 3.89, p < 0.001; children–young adults: z = 0.1, p = 0.995; older adults–young adults: z = -3.84; p<0.001). Furthermore, some maps adopted less clear patterns. Map 1 for example was significantly more present in older adults than in children’s signal, but no other difference among groups reached significance (children–older adults: z = -3.19, p = 0.004; children–young adults: z = -1.26, p = 0.417; older adults–young adults: z = 2.06; p = 0.098). Map 4 showed the same profile, being significantly more present in children than in young adults, while no other significant differences were emphasized (children–older adults: z = 2.05, p = 0.1; children–young adults: z = 2.75, p = 0.016; older adults–young adults: z = 0.76; p = 0.729). Finally, the model failed to highlight differences in the maps involved among the conditions (χ^2^(10) = 5.27; p = 0.872).

In children, none of the maps present on the stimulus-aligned signal explained the Stroop effect, although the tANOVA analysis showed a significant change in topography in this time-window (Fig 3). Regarding the young adults group, longer latencies in the incongruent condition could not be explained by a particular microstate map in the stimulus-aligned signal, since only Map 5 showed significantly increased durations in the congruent condition (t(845) = 3.57; p = 0.001). A longer duration in the congruent condition could be the consequence of an additional map, compressing the time allocated to Map 5, however, no significant interaction was noted between the maps and conditions (χ^2^(10) = 7.38; p = 0.689). A later onset in the incongruent condition could also explain the effect, though, no significant interaction between conditions, maps and age groups were noted (χ^2^(20) = 11.85; p = 0.921), nor an interaction between conditions and maps (χ^2^(10) = 8.24; p = 0.605). A significant effect in the stimulus-aligned signal for young adults is in line with the waveform statistics, while no effect were highlighted on the tANOVA. In the older adults group, Map 1, present from 230ms up to the end of the time window was significantly longer in the incongruent condition (t(366) = -4.81; p<0.001), as highlighted by the tANOVA. Fig 4 illustrates very well this very pronounced effect. For all the age groups. as presented in the detailed results (S5–S8 Tables), some other microstate maps highlighted significant difference but were present in less than 50% of the subjects and were not included on the fitting on the grand average shown in Fig 4, leading to probably unreliable results, therefore, the results were not interpreted.

On the response-aligned signal, when considering the results of the microstates analysis presented in Fig 4, children tend to have different microstate maps than young and older adults. Indeed, Maps 8 (children–older adults: z = 3.8, p<0.001; children–young adults: z = 3.65; p<0.001; older adults–young adults: z = -0.18; p = 0.981) and 9 (children–older adults: z = 4.41, p<0.001; children–young adults: z = 4.72; p<0.001; older adults–young adults: z = 0.43; p = 0.902), the two maps sequentially occurring on the children’s response-aligned signal (Fig 4), were significantly more characteristic of the children group. Despite what is shown on Fig 4, only Map 7 was specific to the two adult groups (children–older adults: z = -4.37; p<0.001; children–young adults: z = -5.45; p<0.001; older adults–young adults: z = -1.65; p = 0.225) and Map 10 did not show any significant effects on the presence (children–older adults: z = -1.95, p = 0.124, children–young adults: z = -1.58, p = 0.254; older adults–young adults: z = 0.39, p = 0.92).

Regarding duration of the microstate maps, results were interpreted based on the maps present on Fig 4 and in accordance to the percentage of presence of each microstate map presented in S2 Fig. As for the stimulus-aligned signal, the statistical model failed to highlight a significant difference of involvement of the maps among the conditions (χ^2^(6) = 9.19; p = 0.163). In the children group, Map 9, i.e. the microstate map occurring right before the onset of the production, failed to highlight a significant difference (t(563) = -1.86, p = 0.153) despite the fact that Map 8 (the farer microstate map from the articulation) showed a significantly reduced duration in the incongruent condition (t(563) = 6.36, p<0.001) and significant tANOVA results was highlighted in the pre-articulatory period. Regarding both adult groups, Map 10, occurring right before the onset of the articulation, lasted respectively longer and shorter in the young (t(289) = -3.13; p = 0.005) and older adult groups (t(289) = 2.93; p = 0.01). The tANOVA as well as the waveforms statistics were in line with the results of the microstates analysis for the older adults group. The young adults group showed an effect around -400ms before the onset of the articulation, but no significance in the pre-articulatory time window were noted. Map 7, which covers the signal from -520 to ~200ms before the articulation onset did not show any effect of condition (t(289) = -0.26; p = 0.963). As for stimulus-aligned results, some other maps highlighted differences between the congruent and incongruent conditions, but given their rare presence in some age groups, the results in these age groups were not interpreted.

On the extended group of older/old adults, the results were very similar. On the stimulus-aligned signal, we still observe a strong main effect of Maps (χ^2^(5) = 117.12; p<0.001); no main effect of condition (χ^2^(2) = 0.24; p = 0.887) and a significant interaction between the two (χ^2^(10) = 59.46; p<0.001). Regarding the post-hoc decomposition of the effect, as reported in the paper, Map 1 still significantly lasts longer in the incongruent condition (t(521) = 3.13; p = 0.005). In the response aligned signal, we report a significant main effect of maps (χ^2^(3) = 103.82; p<0.001), a significant effect of conditions (χ^2^(2) = 15.96; p<0.001) and a significant interaction between the two factors (χ^2^(5) = 136.03; p<0.001). As a reminder, we reported above that in older adults, Map 10 was significantly shorter in the incongruent condition compared to the congruent one. By adding 15 old adults to the older adults group, this effect is now only marginally significant (t(347) = 2.249; p = 0.0646). However, a shortening of Map 10 in the incongruent condition is backed-up by a strongly significant lengthening of Map 7 in this condition (t(347) = -7.59; p<0.001).

#### 3.2.4 Source localization of the microstates

**Fig 5:**
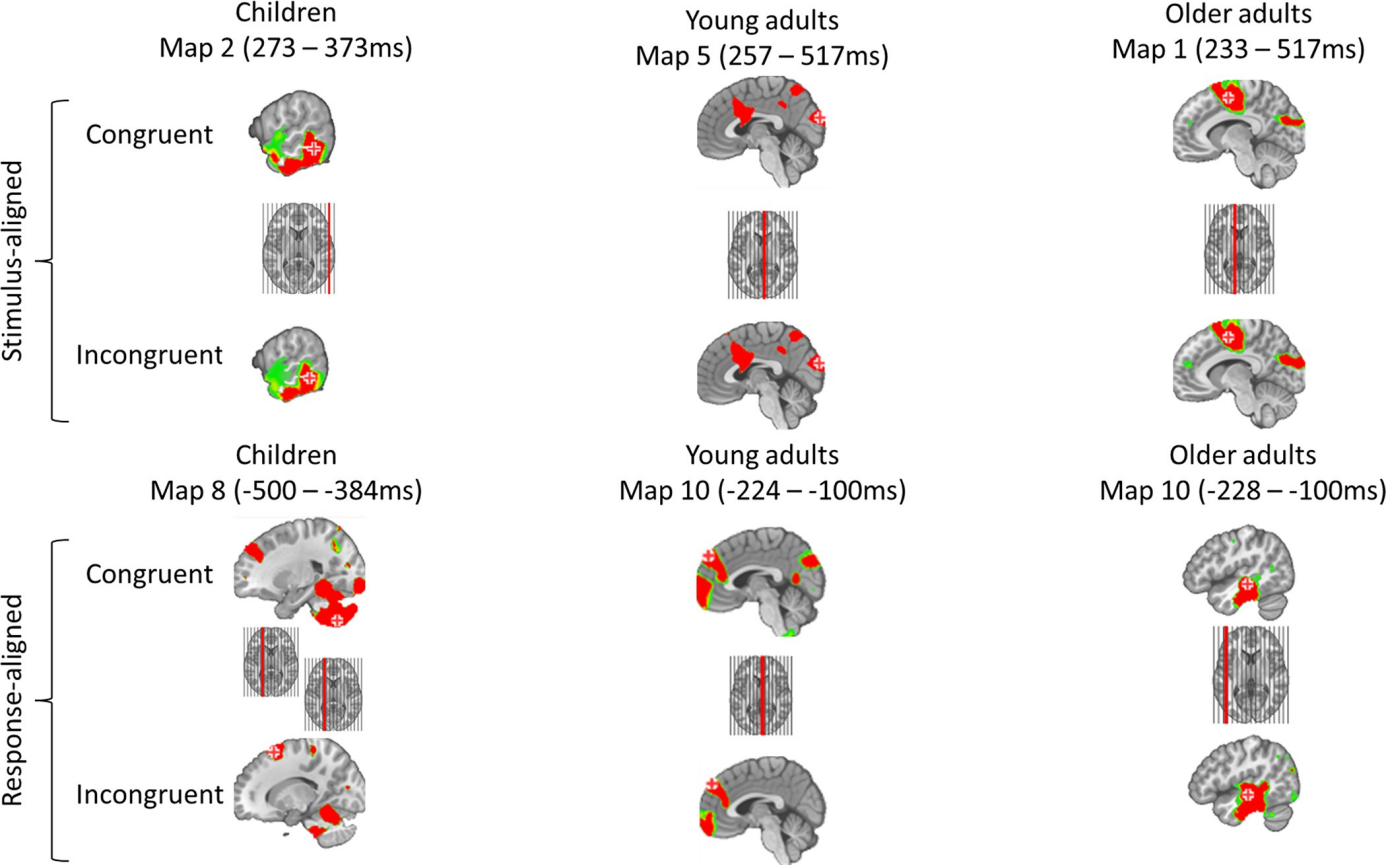
Sources relative to the timing of the microstates disentangling groups or conditions in regards of the Stroop effect. Red cross indicates the maximum of activation.

Given the large number of groups and conditions included in the present study, to guide interpretation of the topographical results, a source localization of the signal underlying the most relevant microstates was performed. The source localization procedure was performed on the grand averages, based on the timing of the microstates maps bearing the differences among groups or conditions (Fig 5). In the stimulus-aligned signal, Map 2 appeared to be specific to the children group but did not show a significant difference between the congruent and incongruent conditions. This map reflects mainly activations in the right posterior middle temporal gyrus. In the young adults group, Map 5 showed a modulation between the congruent and incongruent condition (shorter in the incongruent condition). This microstate map was related to a maximum peak of activation in the posterior occipital cortex, but also in the anterior midcingulate cortex for both congruent and incongruent conditions. Regarding the older adults group, Map 1 seems to last exaggeratedly longer in the incongruent condition. This particular map was underlid by activations in the posterior midcingulate cortex as well as in the fronto-posterior areas.

Regarding the response-aligned signal, children did not highlight an effect of the last microstate map before the onset of the articulation but a modulation of the preceding microstate has been underlined. This earlier microstate (Map 8) showed globally the same brain networks but with different maxima of activation between the congruent and incongruent conditions. In the congruent condition, the activation peak is located in the cerebellum, including activations in the left dorsolateral prefrontal cortex (DLPFC), while a different site of the DLPFC was activated in the incongruent condition, which happened to bear the maximum activation. In young adults, an effect has been highlighted during the timing of activation of Map 10. This map was related to an activation of a fronto-central region, extended to the ACC. Regarding the older adults group, the same microstate map as the one explaining the Stroop effect in young adults showed a modulation (longer in the congruent condition). This map showed the same brain regions as the one highlighted in young adults but with a maximum activation in the left superior temporal sulcus, extending to the left inferior temporal gyrus. In the congruent condition, the left site held the maximum activation while in the incongruent condition, the peak transferred to the right side, involving a wider activation zone.

## 4. Discussion

The present study aimed at understanding how the Stroop effect with its two levels of conflict (perception- and resolution-based conflicts) evolves with age, since latencies are longer for children and older adults. The present results confirmed the larger latencies in both children and older adults reported in previous studies, but the ERP results indicate different underlying processes in development and aging. In the following, we will first briefly comment the behavioral results before digging separately the issues related to development and aging.

### 4.1. Behavioral results

The behavioral results supported the expectations, showing a strong Stroop effect on the latencies for each group while the measurement of accuracy did not highlight clear effects. Indeed, even though the congruent condition was less prone to errors than the incongruent one, no modulation across the age groups was emphasized. Across the three conditions, children were the group showing the largest error rate despite the most narrow age range. Regarding older adults, even though the difference was not significant, it is worthy to note that this group was more accurate compared to their young counterparts, which could translate a change in the speed-accuracy tradeoff between the two groups. Young adults might be more focused on rapidity to provide a swift response while older adults might rather favor accuracy. This might be the first argument claiming that aging is not maturation in reverse [28]. To explore more precisely how maturation and aging differ, topographical analyses were performed on the task to help going beyond the limitations of behavioral and classical ERP methods.

### 4.2. From childhood to adulthood

Specifically in development, it has been clearly established that children show lower performances. This leads to an enhanced Stroop effect on latencies and accuracy of the responses [32]. From a brain perspective, literature tended to explain these lower performances by immature brain networks. In particular, studies claim that the N400 component, underlying the conflict detection processes is in place early (6–7 years old) while conflict resolution processes get matured at about 12 years old [34]. In the present study, we aimed at clarifying what exactly means “immature brain networks”. We aimed at clarifying if some additional processes are required? Are the processes taking longer? Are they different compared to a young adults group for the same time window? The results suggest that these hypotheses are not mutually exclusive. Indeed, Map 2, present from 269 to 377ms on the stimulus-aligned signal, appeared to be specific to children. This Map 2 appeared following the N170 component, which seems common across groups (Map 3). However, the time period of Map 2 does not seem to participate in the interference effect since this microstate did not last significantly longer nor significantly shorter in the congruent versus the incongruent conditions. Seemingly, the process involved in the P300 component is still immature in children [85]. Based on the time course of this map, it may be related to reading [86] or to lexical selection processes [87], or both. The spatial localization of the signal in this time window showed a maximal activation in the right posterior middle temporal gyrus. This regions has rarely been associated to the semantic processing in young adults [88]. Furthermore, other activation sites occurring simultaneously within this map included the bilateral temporal poles, known to be related to semantic processing [89], as well as left insular regions. The latter have been strongly associated to semantic processing [90], as well as automatic word generation [91]. A right hemisphere activation might occur to compensate the still immature left temporal regions, strongly associated with language production and perception. Alternatively, a change in the same temporality has been proposed in the aging literature, stating that the P300 was modulated between young and older adults [15, 20, 22]. As described below, the authors attributed this modulation to a preparatory process related to the attentional functioning aiming at facilitating the processing of the conflict.

On top of this additional microstate map, in the children age group, none of the microstate maps from the stimulus-aligned signal showed increased duration in the incongruent condition, even Map 4 which represents the N400 time window. It is noteworthy to mention that Map 4 was significantly more often identified in the children and older adults groups’ signal. This microstate map might be related to the level of available fluid intelligence. The present study did not include a specific measure of fluid intelligence to corroborate this statement but future studies might be interested in highlighting a biomarker of lowering fluid intelligence on ERP data using microstates analysis.

It is noteworthy to mention that In the young adults group, the microstate map underpinning the N400 component was significantly shorter in the incongruent condition. Moreover, even though waveform analysis showed the classically described N400 effect, the tANOVA did not highlight any significant effect in this time period. The incongruency of this result will be discussed below. However, a shorter conflict detection process in the incongruent condition could lead to several conclusions. It might be possible that to avoid longer reaction times, conflict detection processes are shortened to leave the floor to conflict-resolution processes. On the other hand, the detection of a conflict might be faster when an obvious conflict appears, such as the one experienced in the Stroop task. In the absence of a conflict as when facing a congruent trial, thorough monitoring mechanisms might be involved to ensure that no conflict is present. In any case, the longer latencies observed in the incongruent condition can be explained by an increased duration of the last microstate before the onset of the articulation (conflict-resolution time window), observable on the response-aligned signal. The Stroop effect in young adults can then be explained by a reconfiguration of the conflict detection processes and a conflict cost mainly carried by the conflict resolution processes.

As presented in Fig 4, in the children and young adults group, the microstate maps explaining the N400 time window are dissimilar, implying that children show immature brain processes. To better understand in what the topographies differed, the source localization procedure revealed that the brain networks involved share common areas, such as the ACC, but children showed additional sources, mainly in the orbito-frontal regions. The requirement of additional brain networks should be interpreted as less efficient brain functioning. Indeed, it has been showed that the more trained an ability, the less brain regions involved [92]. In the response aligned signal, the distribution of the different topographic maps is globally similar.

When aligning the epochs to the response onset, in all three age groups, two microstate maps were present. Nevertheless, children tended to show particular microstate maps while the two adults group showed other ones (even though Map 10 did not significantly differ in presence among the groups). It appears then that the dynamic of the organization of the microstates is similar but the networks involved in children are not yet adult-like. Moreover, the tANOVA results support a difference in topographies occurring close to the response onset. The microstates analysis partially supported this results. Indeed, only Map 8, occurring far from the response onset, appeared to be significantly shorter. Based on Fig 4, this could imply a potential difference on the pre-articulatory period which failed to reach significance. As already argued in the literature, the unreliability of the results might point out that age seems to be a poor index of developmental status [93]. Some children in this group might have very adult-like patterns while some other are still closer to younger children, leading to a lower part of variance explained by statistical models. It is noteworthy to mention that although a majority of studies found the same N400 effect as described in the adult literature [16, 12, 19] but some other failed to report this difference in a developmental population [36].

To sum-up the results, it appears that in both children and young adults, the same brain networks are involved in all conditions. The results also suggest that longer latencies in children are probably related to several factors. First, an additional brain network was identified. Based on the timing of the activation as well as the source localization analysis, the latter network is very likely to be related to word perception/production. This additional process might be participating in the longer latencies. Furthermore, as expected, brain networks are still immature in children, who display different topographic maps to explain both the conflict detection and resolution processes. However, these topographies are not dramatically different but rather include additional brain areas, suggesting a less efficient functioning. A discrepancy between the different methods included in the present study has been observed. These discrepancies might be interpreted as an illustration of the large variability of developmental status in 10 to 12 year old children. Furthermore, our results are in line with the one presented in the literature. Indeed, Jongen and Jonkman [34] suggested that the conflict detection processes are in place early in development, but the conflict resolution tend to evolve up to 12 years old. In our results, effects on duration of the microstates were present mainly in the response-aligned signal. However, different topographic maps were highlighted in the N400 time window, suggesting that the brain processes are not still fully similar between 10–12 years old children and young adults.

### 4.3. Aging

Even though from the point of view of latencies older adults tend to show the same increased response time regarding the interference effect as children, the precise mechanisms explaining the longer latencies in this group remain to be explored. Children showed underdeveloped specific brain networks but older adults might show a different pattern such as similar networks differing only in duration, then favoring the general slowing hypothesis.

Once again, the results favored the hypothesis of identical mechanisms involved in congruent and incongruent conditions, already discussed above. Regarding stimulus-aligned signal, young and older adults show a wide microstate map involved from 220 up to 520ms post-stimulus onset, underpinning the N400 time window. However, these two maps are different between young and older adults (Map 5 for young adults and Map 1 for older adults). Despite these differences, probably related to the different configurations of the iso-electric lines, they share the same electrode of maximum and minimum amplitude. The source reconstruction procedure also pointed out to a relatively similar network of structures, involving the left DLPFC as well as the cingulate cortex. The contribution of these two maps was also different between the two adult groups. As already stated above, this map appeared to be shorter in the incongruent condition, and then did not participate to the longer latencies. However, in older adults, Map 1 appeared to be dramatically longer in the incongruent condition. Interestingly, this map starts in the temporal window of the P300 component, which has been shown to be modulated in aging for the Stroop task [15, 20, 22]. In particular, this corroborates the findings of West and Alain [22], who revealed that the effect of aging was visible on the ERP signal from 300ms up to the response onset.

In the response-aligned signal, both adult groups show a modulation of Map 10 (right before the articulation onset). In young adults, this map explains slower latencies in the incongruent congruent condition compared to the congruent one, while older adults showed the opposite pattern. The source localization analysis showed activation in the same sites but with different maxima. Young adults display a reliable activation of the ACC, the right DLPFC and orbito-frontal regions, which is in accordance with the literature [4, 16, 94]. Older adults’ signal revealed the same regions but with an activation peak observed on the left superior temporal sulcus, extending to the left inferior temporal gyrus. This region has been related to word-form encoding and monitoring processes [87]. It might be possible that with age, the interference processing uses more semantic features than executive functions, as suggested by Spreng and Turner [95]. They hypothesized that since at first, children can rely only on fluid intelligence and since in aging, adults tend to show a higher crystallized intelligence but lower fluid intelligence abilities, architectural changes along the lifespan experience a transition from fluid to crystallized intelligence engaged to perform cognitive tasks. This theory could explain the changes observed in the source localization analysis, however interference effect (incongruent vs. congruent trials) within each age group remains to be discussed.

In the conflict resolution time window, an increased duration of the map preceding the articulation was noted (Map 10) in the congruent condition. The most likely interpretation would be that the compression of Map 10 is a compensatory mechanism aiming at maintaining the performances without sacrificing the accuracy. Acoustic investigations combined with EEG analyses on participants’ responses analyzing the differences in properties of the oral production would be an interesting and novel approach to confirm this interpretation.

As a reminder, it was assumed that results favoring the general slowing hypothesis would show the same brain networks involved in both young and older adults, differing only in duration. In case of differences in microstate maps explaining the same time window or additional processes involved, then the worsening of performances in aging would be better explained by specific impairments related to aging. Based on the results presented above, in opposition to children, no additional map was noted in older adults compared to young adults. However, in the stimulus-aligned results the time window of the N400 component was explained by different microstate maps. Nevertheless, those maps share some topographical similitudes and point out very similar brain networks. Given the dramatically longer duration of the microstate map observed in the older adults signal, this difference in topographical state should be interpreted as a cognitive decline. Concerning conflict-resolution, the results are more homogeneous between the two adult groups. The same microstate map are highlighted, although a slight reconfiguration might have occurred when considering the source localization analysis. As detailed above, older adults tend to rely on language-related areas to perform the Stroop task. This finding is in line with the semanticization hypothesis [95], implying that older adults rather use crystallized intelligence to compensate for a loss in the fluid intelligence abilities. In aging, then, it appears that the conflict detection processes, which rely mainly on attentional and executive resources decline, while the conflict resolution processes seems spared, possibly thanks to a compensatory mechanism (semanticization), involving a reconfiguration of the brain networks engaged.

### 4.4 Limitations

A few limitations need to be addressed regarding the present study. First, the sample size seemed reasonable given that previous studies included from 8 to 25 participants in average [12, 14–17, 20].To ensure that a limited sample size did not lower the reliability of our results, we performed the main within-group analyses (tANOVA and duration of the microstates) on an extended group of older/old adults and found very similar results. This provides us confidence in our results despite having only 17 to 18 participants per group. Moreover, enrolling older adults aged less than 70 years old might not be fully representative of the population of old adults recruited in the majority of the studies investigating aging. Nevertheless, this group was preferred to elderly subjects to avoid cognitive decline and selection bias related to the fact that only the more cognitively performant older persons agreed to participate to neuroscientific studies. Furthermore, when adding old adults to the older adults group provided similar results. Finally, education level differed significantly between the two adult groups, which could also participate in the effects reported regarding the individual features of the microstate analyses. It is noteworthy to mention that in average, young adults had only two more years of education compared to older adults. Even though the present results bring some clarification on the evolution of the Stroop effect, several issues remain open. Undeniably, it is still difficult to attribute a precise process to each of the brain networks highlighted by the microstates analyses. Future studies might need to replicate the result and compare tasks with different involvement of attentional abilities and of cognitive control. It seems also important to better understand the difference between verbal and manual tasks and how it relates to interference. Since differences were observed on the last stages of verbal responses, the divergent results with manual ones could be exploited in a direct comparison to clarify the underlying mental processes.

## 5. Conclusion

The present study aimed at understanding if longer latencies in children or older adults compared to young adults could be attributable to the suboptimal functioning of a specific process, the addition of a specific process or a slowdown of all the involved processes. As discussed above, the results in children showed that the brain networks involved were substantially different from those engaged by the young adults group on both conflict detection and resolution. Longer latencies in children could be explained by an additional microstate map occurring at about 300ms post stimulus onset and probably related to word perception or production, as well as a lengthening of the microstate maps involved in the conflict resolution time window. In other words, this group show immature brain processes and additional ones, reflecting immature brain functioning. In aging, results tell a different story. A much larger cost of conflict detection processes was noted. Moreover, this process was topographically different than the one observed in young adults, implying a decline of the network’s efficiency. While the Stroop effect in young adults was mainly explained by an increased duration of the conflict resolution processes, no such effect was observed in the older adults’ signal. Older adults then tend to show relatively similar brain networks as the one observed in young adults but latencies are slowed down by a slowing and alteration of the brain networks involved in conflict detection. Regarding conflict resolution, older adults might rely more on crystallized intelligence than fluid one. The present study allowed to better characterize why children and older adults show slower latencies at the Stroop task, opening the door to more papers using topographical analyses to explore changes in the EEG signal across the lifespan.

## Supporting information

S1 Fig Mass univariate tests on the waveform for each tested effect. The upper plots row represents the stimulus-aligned results while the bottom row represents the waveforms 500ms before the response onset. The last 100ms were counted as pre-articulation and were excluded from the analyses. The x axis of each graph represents the time in milliseconds and each electrode is represented on the y axis. The dotted vertical line represents the stimulus onset, and the signal beneath the solid horizontal line represents the tANOVA results specific to each effect.
(TIF)

S2 Fig Bar plot of the presence of the maps for each age group and condition. Values on the y axis represent the number of subjects in the age group for which this map was found, and the percentage represents the proportion of this number of subjects relatively to the number of subjects in the age group.
(TIF)

S3 Fig Bar plots reporting the median duration (for subjects who showed this map in their average) and its standard error for each map per conditions and age groups. (TIF)

S4 Fig Dynamic of the topographies over time for each age group and condition by step of 12ms for the stimulus-aligned data and 16ms for the response-aligned data. Warm colors represents amplitudes higher than the average and cold colors amplitudes lower than the average.
(TIFF)

S5 Fig Plot of the global explained variance in relationship with the number of microstate maps for both stimulus- and response-aligned signal. In Ragu, the optimal number of map is calculated following a cross-validation procedure. The explained variance of the map are presented in both the learning set and the test set.
(TIF)

S1 Table Main effects and interactions results regarding the generalized linear mixed model evaluating differences in presence of the microstate maps among the conditions and age groups on the stimulus-aligned signal. The R command of the model is transcribed on the first row.
(DOCX)

S2 Table Post-hoc decomposition of the model presented in S1 Table. (DOCX)

S3 Table Main effects and interactions results regarding the generalized linear mixed model evaluating differences in presence of the microstate maps among the conditions and age groups on the response-aligned signal. The R command of the model is transcribed on the first row.
(DOCX)

S4 Table Post-hoc decomposition of the model presented in S3 Table. (DOCX)

S5 Table Main effects and interactions results regarding the generalized linear mixed model (Hurdle model) evaluating differences in duration of the microstate maps among the conditions and age groups on the stimulus-aligned signal. The R command of the model is transcribed on the first row.
(DOCX)

S6 Table Post-hoc decomposition of the model presented in S5 Table. (DOCX)

S7 Table Main effects and interactions results regarding the generalized linear mixed model (Hurdle model) evaluating differences in duration of the microstate maps among the conditions and age groups on the response-aligned signal. The R command of the model is transcribed on the first row.
(DOCX)

S8 Table Post-hoc decomposition of the model presented in S7 Table. (DOCX)

S9 Table Main effects and interactions results regarding the generalized linear mixed model evaluating differences in onset time of the microstate maps among the conditions and age groups on the stimulus-aligned signal. The R command of the model is transcribed on the first row.
(DOCX)

S10 Table Post-hoc decomposition of the model presented in S9 Table. (DOCX)

S11 Table Main effects and interactions results regarding the generalized linear mixed model evaluating differences in onset time of the microstate maps among the conditions and age groups on the response-aligned signal. The R command of the model is transcribed on the first row.
(DOCX)

S12 Table Post-hoc decomposition of the model presented in S11 Table. (DOCX)
