# Supplementary figures and images for: The temporal dynamics of the Stroop effect from childhood to young and older adulthood
Source: PLoS One. 2023 Mar 30;18(3):e0256003. doi: 10.1371/journal.pone.0256003 (PMC10062650; doi:10.1371/journal.pone.0256003)

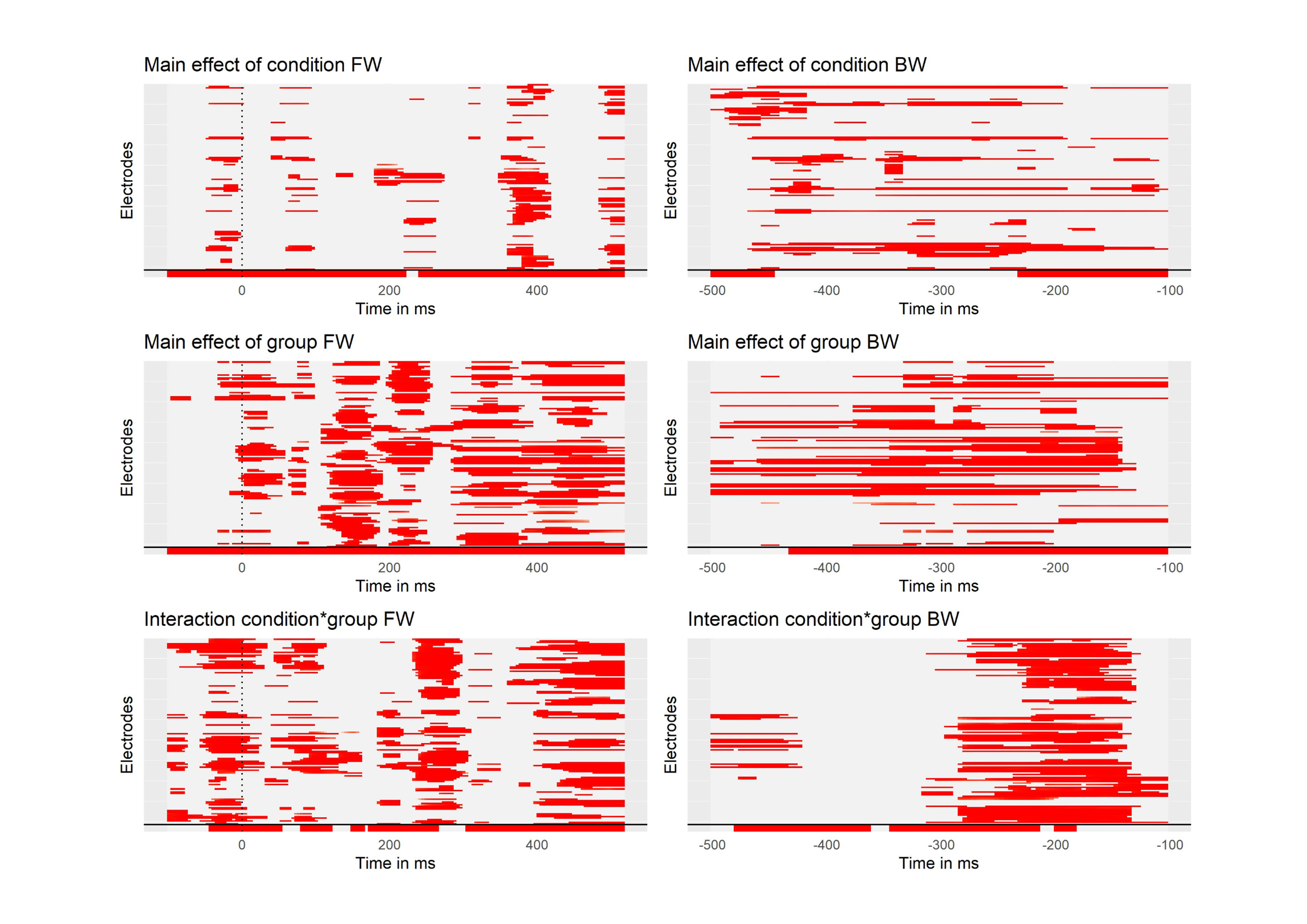

Supplement: S1 Fig — The upper plots row represents the stimulus-aligned results while the bottom row represents the waveforms 500ms before the response onset. The last 100ms were counted as pre-articulation and were excluded from the analyses. The x axis of each graph represents the time in milliseconds and each electrode is represented on the y axis. The dotted vertical line represents the stimulus onset, and the signal beneath the solid horizontal line represents the tANOVA results specific to each effect. (TIF) [file pone.0256003.s001.tif]

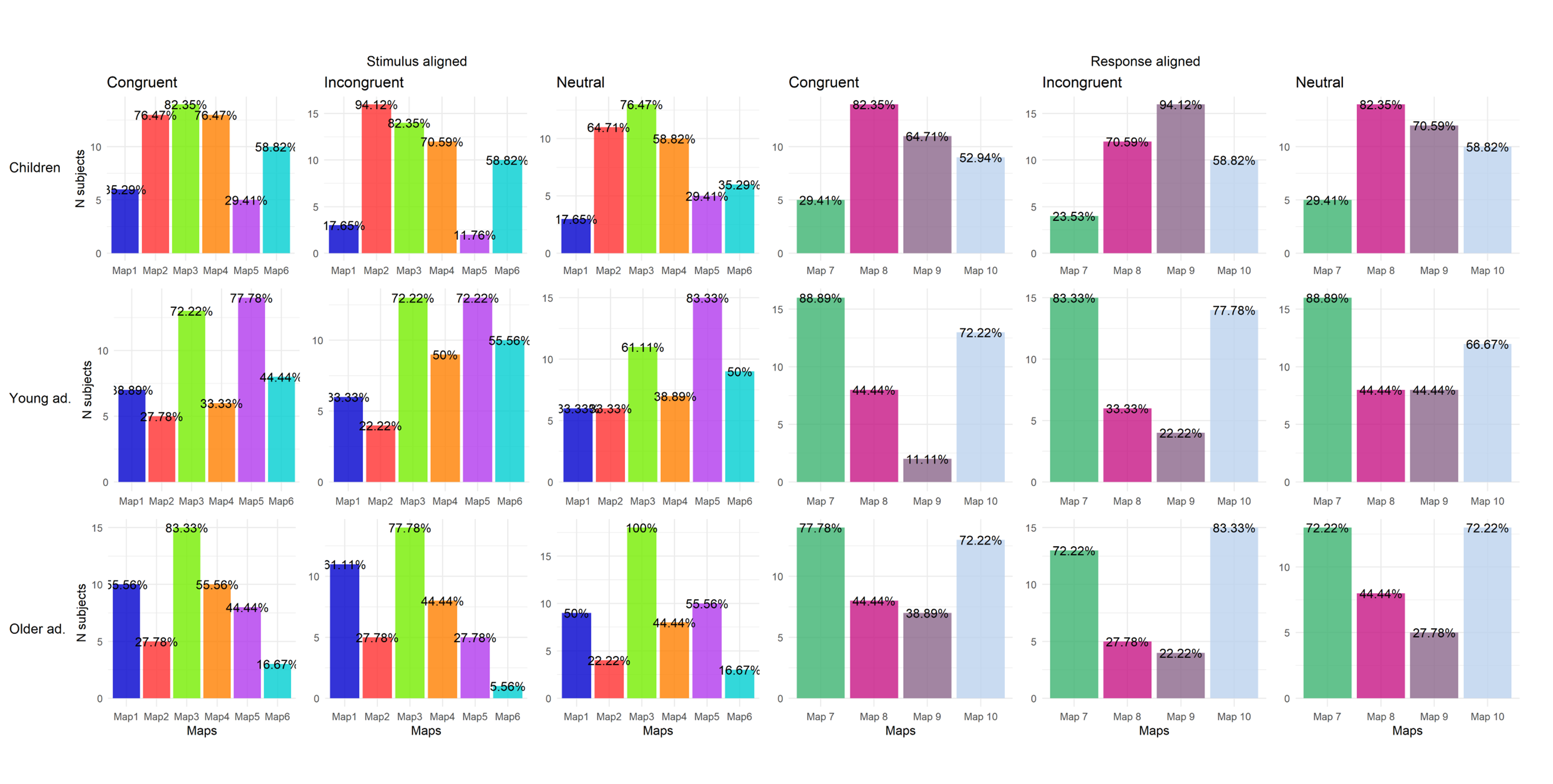

Supplement: S2 Fig — Values on the y axis represent the number of subjects in the age group for which this map was found, and the percentage represents the proportion of this number of subjects relatively to the number of subjects in the age group. (TIF) [file pone.0256003.s002.tif]

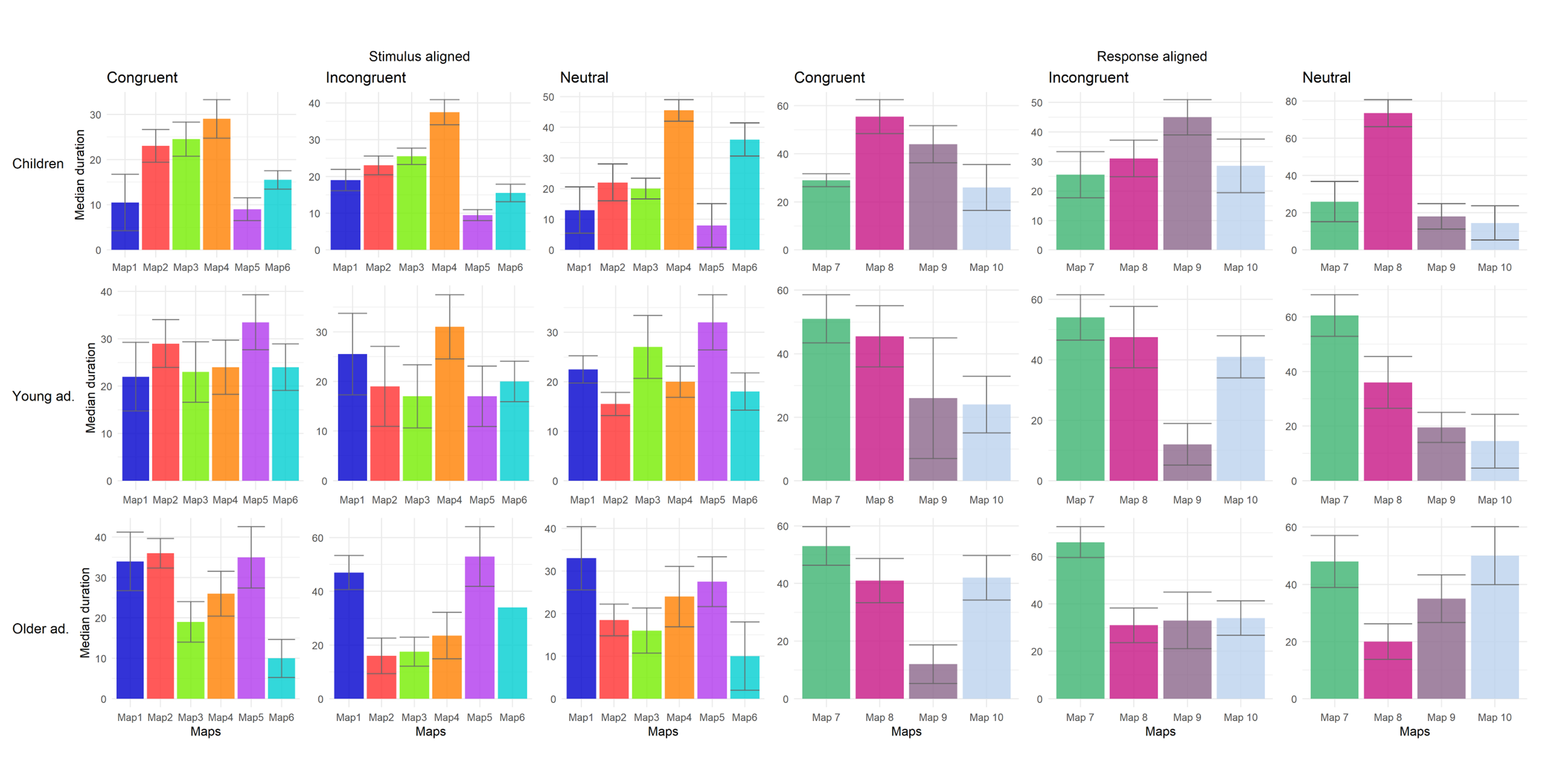

Supplement: S3 Fig — (TIF) [file pone.0256003.s003.tif]

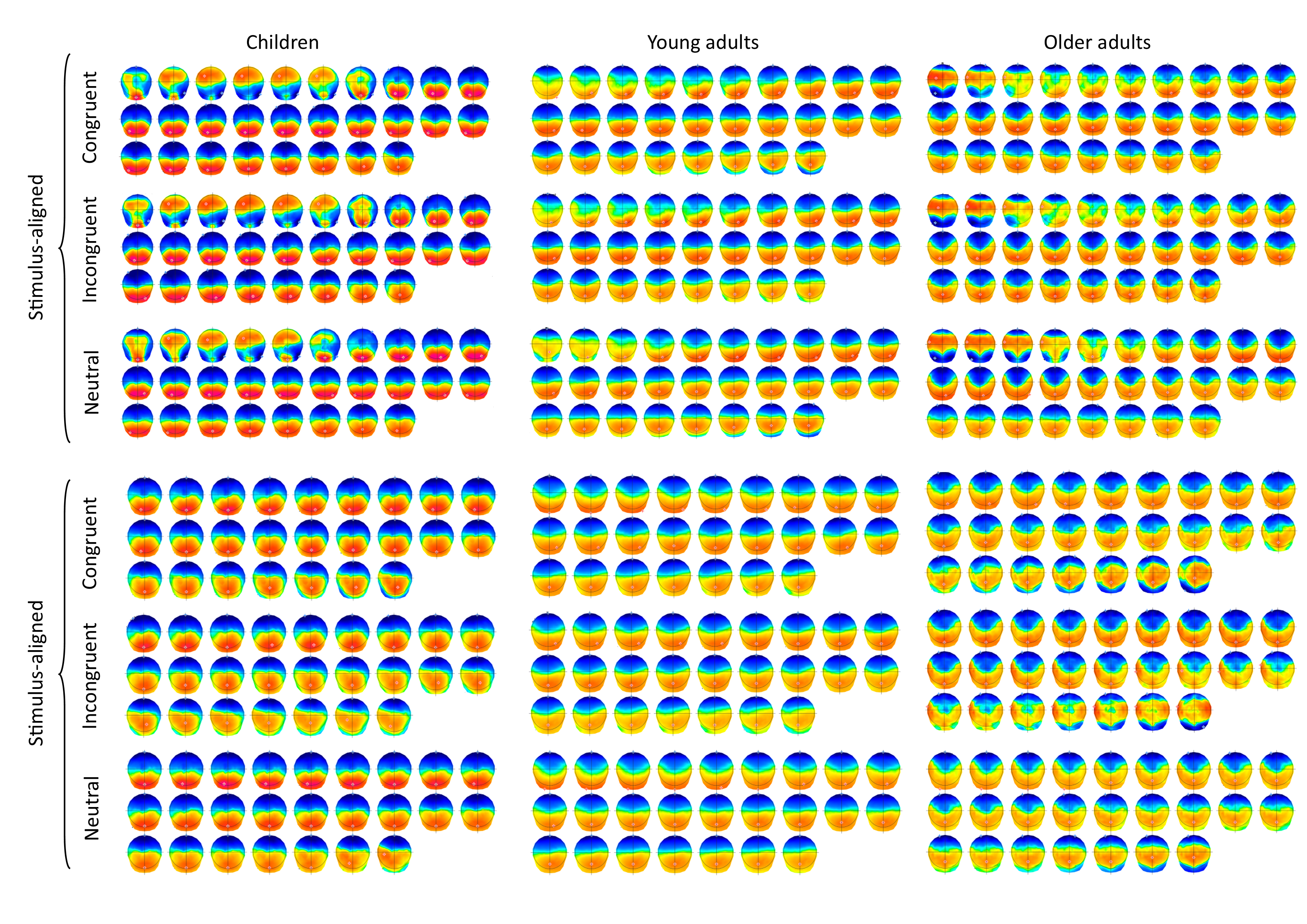

Supplement: S4 Fig — Warm colors represents amplitudes higher than the average and cold colors amplitudes lower than the average. (TIFF) [file pone.0256003.s004.tiff]

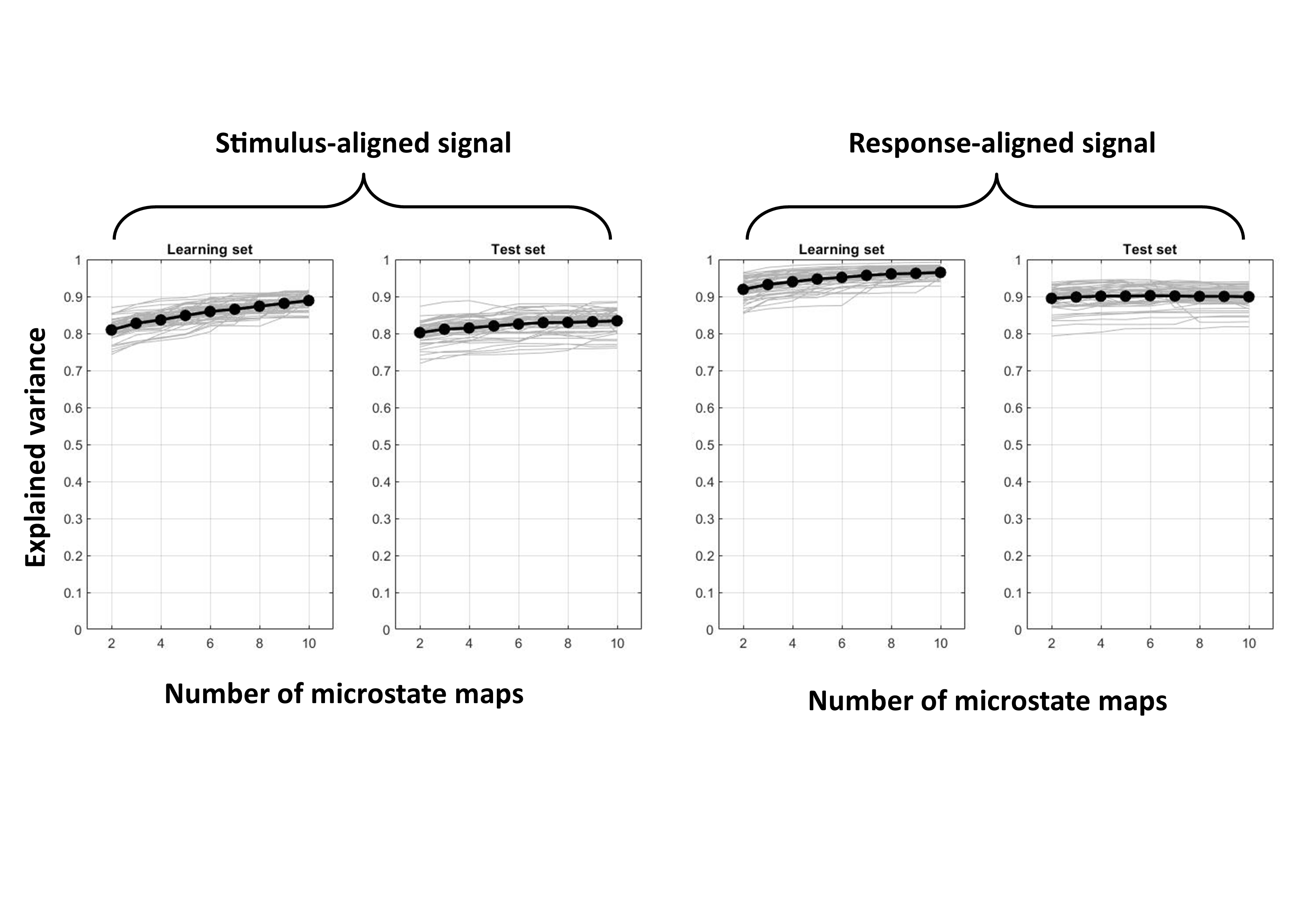

Supplement: S5 Fig — In Ragu, the optimal number of map is calculated following a cross-validation procedure. The explained variance of the map are presented in both the learning set and the test set. (TIF) [file pone.0256003.s005.tif]
