# Supplementary material for: The temporal dynamics of the Stroop effect from childhood to young and older adulthood
Source: PLoS One. 2023 Mar 30;18(3):e0256003. doi: 10.1371/journal.pone.0256003 (PMC10062650; doi:10.1371/journal.pone.0256003)
Supplement: S1 Table — The R command of the model is transcribed on the first row. (DOCX) [file pone.0256003.s006.docx]

| ***Model:*** *glmer(presence ~ Maps*age groups + Maps*conditions + (1\|Subjects ID), family = “binomial”, data = data stimulus-aligned, glmerControl(optimizer = “bobyqa”))* | | | |
| --- | --- | --- | --- |
| **Effect** | **Chisq** | **Df** | **Pr(>Chisq)** |
| Maps | 51.662 | 5 | <0.001 |
| Age group | 2.476 | 2 | 0.29 |
| Conditions | 1.076 | 2 | 0.584 |
| Maps*age group | 101.544 | 10 | <0.001 |
| Maps*conditions | 7.376 | 10 | 0.69 |
