# Supplementary material for: The temporal dynamics of the Stroop effect from childhood to young and older adulthood
Source: PLoS One. 2023 Mar 30;18(3):e0256003. doi: 10.1371/journal.pone.0256003 (PMC10062650; doi:10.1371/journal.pone.0256003)
Supplement: S2 Table — (DOCX) [file pone.0256003.s007.docx]

| **Contrast** | **Maps** | **Estimate** | **SE** | **Df** | **z.ratio** | **P.Value** |
| --- | --- | --- | --- | --- | --- | --- |
| Children - Older Adults | Map1 | -1.453 | 0.455 | Inf | -3.193 | 0.004 |
| Children - Young Adults | Map1 | -0.582 | 0.461 | Inf | -1.261 | 0.417 |
| Older Adults - Young Adults | Map1 | 0.871 | 0.423 | Inf | 2.062 | 0.098 |
| Children - Older Adults | Map2 | 2.423 | 0.486 | Inf | 4.982 | <0.001 |
| Children - Young Adults | Map2 | 2.33 | 0.483 | Inf | 4.829 | <0.001 |
| Older Adults - Young Adults | Map2 | -0.093 | 0.459 | Inf | -0.203 | 0.978 |
| Children - Older Adults | Map3 | -0.505 | 0.557 | Inf | -0.907 | 0.636 |
| Children - Young Adults | Map3 | 0.644 | 0.481 | Inf | 1.337 | 0.374 |
| Older Adults - Young Adults | Map3 | 1.149 | 0.521 | Inf | 2.205 | 0.07 |
| Children - Older Adults | Map4 | 0.887 | 0.433 | Inf | 2.051 | 0.1 |
| Children - Young Adults | Map4 | 1.202 | 0.436 | Inf | 2.754 | 0.016 |
| Older Adults - Young Adults | Map4 | 0.315 | 0.415 | Inf | 0.757 | 0.729 |
| Children - Older Adults | Map5 | -0.934 | 0.461 | Inf | -2.025 | 0.106 |
| Children - Young Adults | Map5 | -2.598 | 0.502 | Inf | -5.17 | <0.001 |
| Older Adults - Young Adults | Map5 | -1.664 | 0.463 | Inf | -3.595 | 0.001 |
| Children - Older Adults | Map6 | 2.001 | 0.515 | Inf | 3.886 | <0.001 |
| Children - Young Adults | Map6 | 0.041 | 0.418 | Inf | 0.099 | 0.995 |
| Older Adults - Young Adults | Map6 | -1.96 | 0.511 | Inf | -3.839 | <0.001 |
