# Supplementary material for: The temporal dynamics of the Stroop effect from childhood to young and older adulthood
Source: PLoS One. 2023 Mar 30;18(3):e0256003. doi: 10.1371/journal.pone.0256003 (PMC10062650; doi:10.1371/journal.pone.0256003)
Supplement: S3 Table — The R command of the model is transcribed on the first row. (DOCX) [file pone.0256003.s008.docx]

| ***Model:*** *glmer(presence ~ Maps*age groups + Maps*conditions + (1\|Subjects ID), family = “binomial”, data = data response-aligned, glmerControl(optimizer = “bobyqa”))* | | | |
| --- | --- | --- | --- |
| Effect | Chisq | Df | Pr(>Chisq) |
| Maps | 19.222 | 3 | <0.001 |
| Age group | 0.44 | 2 | 0.803 |
| Conditions | 0.526 | 2 | 0.769 |
| Maps*age_group | 88.46 | 6 | <0.001 |
| Maps*conditions | 9.197 | 6 | 0.163 |
