# Supplementary material for: The temporal dynamics of the Stroop effect from childhood to young and older adulthood
Source: PLoS One. 2023 Mar 30;18(3):e0256003. doi: 10.1371/journal.pone.0256003 (PMC10062650; doi:10.1371/journal.pone.0256003)
Supplement: S4 Table — (DOCX) [file pone.0256003.s009.docx]

| **Contrast** | **Maps** | **Estimate** | **SE** | **Df** | **z.ratio** | **P.Value** |
| --- | --- | --- | --- | --- | --- | --- |
| Children - Older Adults | Map_7 | -2.057 | 0.489 | Inf | -4.208 | <0.001 |
| Children - Young Adults | Map_7 | -3.077 | 0.565 | Inf | -5.45 | <0.001 |
| Older Adults - Young Adults | Map_7 | -1.02 | 0.55 | Inf | -1.855 | 0.152 |
| Children - Older Adults | Map_8 | 1.881 | 0.494 | Inf | 3.809 | <0.001 |
| Children - Young Adults | Map_8 | 1.801 | 0.494 | Inf | 3.647 | 0.001 |
| Older Adults - Young Adults | Map_8 | -0.08 | 0.448 | Inf | -0.178 | 0.983 |
| Children - Older Adults | Map_9 | 2.205 | 0.499 | Inf | 4.415 | <0.001 |
| Children - Young Adults | Map_9 | 2.42 | 0.512 | Inf | 4.73 | <0.001 |
| Older Adults - Young Adults | Map_9 | 0.215 | 0.482 | Inf | 0.446 | 0.896 |
| Children - Older Adults | Map_10 | -0.849 | 0.479 | Inf | -1.773 | 0.179 |
| Children - Young Adults | Map_10 | -0.872 | 0.481 | Inf | -1.811 | 0.166 |
| Older Adults - Young Adults | Map_10 | -0.022 | 0.5 | Inf | -0.044 | 0.999 |
