# Supplementary material for: The temporal dynamics of the Stroop effect from childhood to young and older adulthood
Source: PLoS One. 2023 Mar 30;18(3):e0256003. doi: 10.1371/journal.pone.0256003 (PMC10062650; doi:10.1371/journal.pone.0256003)
Supplement: S5 Table — The R command of the model is transcribed on the first row. (DOCX) [file pone.0256003.s010.docx]

| ***Model****: glmmTMB(duration ~ Maps*conditions*age groups + (1\|Subjects ID), data = data stimulus-aligned, ziformula = ~ Maps*conditions*age groups, family = truncated_poisson )* | | | |
| --- | --- | --- | --- |
| **Effects** | **Chisq** | **Df** | **Pr(>Chisq)** |
| Maps | 239.079 | 5 | <0.001 |
| Conditions | 9.163 | 2 | 0.01 |
| Age group | 1.493 | 2 | 0.474 |
| Maps*conditions | 58.009 | 10 | <0.001 |
| Maps*age group | 313.043 | 10 | <0.001 |
| Conditions*age group | 51.509 | 4 | <0.001 |
| Maps*conditions*age group | 170.586 | 20 | <0.001 |
