# Supplementary material for: The temporal dynamics of the Stroop effect from childhood to young and older adulthood
Source: PLoS One. 2023 Mar 30;18(3):e0256003. doi: 10.1371/journal.pone.0256003 (PMC10062650; doi:10.1371/journal.pone.0256003)
Supplement: S6 Table — (DOCX) [file pone.0256003.s011.docx]

| Contrast | Maps | Age Group | Estimate | SE | df | t.ratio | p.value |
| --- | --- | --- | --- | --- | --- | --- | --- |
| C - I | Map1 | Children | 0.112 | 0.171 | 366 | 0.659 | 0.788 |
| C - N | Map1 | Children | 0.077 | 0.169 | 366 | 0.454 | 0.892 |
| I - N | Map1 | Children | -0.036 | 0.189 | 366 | -0.189 | 0.981 |
| C - I | Map2 | Children | 0.063 | 0.078 | 366 | 0.813 | 0.695 |
| C - N | Map2 | Children | -0.171 | 0.081 | 366 | -2.124 | 0.086 |
| I - N | Map2 | Children | -0.235 | 0.077 | 366 | -3.032 | 0.007 |
| C - I | Map3 | Children | 0.011 | 0.073 | 366 | 0.146 | 0.988 |
| C - N | Map3 | Children | 0.17 | 0.078 | 366 | 2.194 | 0.073 |
| I - N | Map3 | Children | 0.16 | 0.078 | 366 | 2.052 | 0.102 |
| C - I | Map4 | Children | -0.155 | 0.07 | 366 | -2.213 | 0.07 |
| C - N | Map4 | Children | -0.487 | 0.069 | 366 | -7.04 | <0.001 |
| I - N | Map4 | Children | -0.332 | 0.068 | 366 | -4.889 | <0.001 |
| C - I | Map5 | Children | 0.353 | 0.266 | 366 | 1.328 | 0.38 |
| C - N | Map5 | Children | -0.288 | 0.169 | 366 | -1.699 | 0.207 |
| I - N | Map5 | Children | -0.64 | 0.258 | 366 | -2.486 | 0.036 |
| C - I | Map6 | Children | -0.051 | 0.11 | 366 | -0.464 | 0.888 |
| C - N | Map6 | Children | -0.691 | 0.11 | 366 | -6.297 | <0.001 |
| I - N | Map6 | Children | -0.64 | 0.107 | 366 | -5.962 | <0.001 |
| C - I | Map1 | Older Adults | -0.343 | 0.071 | 366 | -4.816 | <0.001 |
| C - N | Map1 | Older Adults | -0.099 | 0.078 | 366 | -1.271 | 0.413 |
| I - N | Map1 | Older Adults | 0.243 | 0.071 | 366 | 3.434 | 0.002 |
| C - I | Map2 | Older Adults | 0.264 | 0.123 | 366 | 2.148 | 0.082 |
| C - N | Map2 | Older Adults | 0.739 | 0.149 | 366 | 4.963 | <0.001 |
| I - N | Map2 | Older Adults | 0.475 | 0.157 | 366 | 3.034 | 0.007 |
| C - I | Map3 | Older Adults | -0.063 | 0.075 | 366 | -0.833 | 0.683 |
| C - N | Map3 | Older Adults | -0.091 | 0.071 | 366 | -1.277 | 0.409 |
| I - N | Map3 | Older Adults | -0.028 | 0.071 | 366 | -0.396 | 0.917 |
| C - I | Map4 | Older Adults | 0 | 0.089 | 366 | -0.002 | 1 |
| C - N | Map4 | Older Adults | -0.073 | 0.087 | 366 | -0.842 | 0.677 |
| I - N | Map4 | Older Adults | -0.073 | 0.091 | 366 | -0.799 | 0.704 |
| C - I | Map5 | Older Adults | -0.055 | 0.088 | 366 | -0.629 | 0.804 |
| C - N | Map5 | Older Adults | 0.319 | 0.081 | 366 | 3.92 | <0.001 |
| I - N | Map5 | Older Adults | 0.374 | 0.091 | 366 | 4.116 | <0.001 |
| C - I | Map6 | Older Adults | -0.79 | 0.238 | 366 | -3.324 | 0.003 |
| C - N | Map6 | Older Adults | -0.184 | 0.213 | 366 | -0.864 | 0.664 |
| I - N | Map6 | Older Adults | 0.606 | 0.235 | 366 | 2.577 | 0.028 |
| C - I | Map1 | Young Adults | -0.14 | 0.107 | 366 | -1.302 | 0.395 |
| C - N | Map1 | Young Adults | 0.036 | 0.111 | 366 | 0.319 | 0.946 |
| I - N | Map1 | Young Adults | 0.175 | 0.113 | 366 | 1.554 | 0.267 |
| C - I | Map2 | Young Adults | -0.115 | 0.141 | 366 | -0.814 | 0.694 |
| C - N | Map2 | Young Adults | 0.585 | 0.146 | 366 | 4.016 | <0.001 |
| I - N | Map2 | Young Adults | 0.701 | 0.15 | 366 | 4.655 | <0.001 |
| C - I | Map3 | Young Adults | -0.055 | 0.075 | 366 | -0.737 | 0.741 |
| C - N | Map3 | Young Adults | -0.208 | 0.075 | 366 | -2.782 | 0.016 |
| I - N | Map3 | Young Adults | -0.153 | 0.075 | 366 | -2.045 | 0.103 |
| C - I | Map4 | Young Adults | -0.315 | 0.103 | 366 | -3.071 | 0.006 |
| C - N | Map4 | Young Adults | 0.154 | 0.118 | 366 | 1.308 | 0.391 |
| I - N | Map4 | Young Adults | 0.469 | 0.103 | 366 | 4.575 | <0.001 |
| C - I | Map5 | Young Adults | 0.25 | 0.07 | 366 | 3.574 | 0.001 |
| C - N | Map5 | Young Adults | -0.072 | 0.063 | 366 | -1.155 | 0.481 |
| I - N | Map5 | Young Adults | -0.322 | 0.068 | 366 | -4.71 | <0.001 |
| C - I | Map6 | Young Adults | 0.182 | 0.099 | 366 | 1.841 | 0.158 |
| C - N | Map6 | Young Adults | 0.153 | 0.101 | 366 | 1.512 | 0.287 |
| I - N | Map6 | Young Adults | -0.03 | 0.1 | 366 | -0.295 | 0.953 |
