# Supplementary material for: The temporal dynamics of the Stroop effect from childhood to young and older adulthood
Source: PLoS One. 2023 Mar 30;18(3):e0256003. doi: 10.1371/journal.pone.0256003 (PMC10062650; doi:10.1371/journal.pone.0256003)
Supplement: S7 Table — The R command of the model is transcribed on the first row. (DOCX) [file pone.0256003.s012.docx]

| ***Model****: glmmTMB(duration ~ Maps*conditions*age groups + (1\|Subjects ID), data = data response-aligned, ziformula = ~ Maps*conditions*age groups, family = truncated_poisson )* | | | |
| --- | --- | --- | --- |
| **Effects** | **Chisq** | **Df** | **Pr(>Chisq)** |
| Maps | 299.451 | 3 | <0.001 |
| Conditions | 7.078 | 2 | 0.029 |
| Age group | 0.734 | 2 | 0.693 |
| Maps*conditions | 60.537 | 6 | <0.001 |
| Maps*age_group | 282.533 | 6 | <0.001 |
| Conditions*age_group | 14.153 | 4 | 0.007 |
| Maps*conditions*age group | 300.027 | 12 | <0.001 |
