# Supplementary material for: The temporal dynamics of the Stroop effect from childhood to young and older adulthood
Source: PLoS One. 2023 Mar 30;18(3):e0256003. doi: 10.1371/journal.pone.0256003 (PMC10062650; doi:10.1371/journal.pone.0256003)
Supplement: S8 Table — (DOCX) [file pone.0256003.s013.docx]

| Contrast | Maps | Age Group | Estimate | SE | df | t ratio | p value |
| --- | --- | --- | --- | --- | --- | --- | --- |
| C - I | Map_7 | Children | -0.264 | 0.131 | 289 | -2.021 | 0.109 |
| C - N | Map_7 | Children | -0.258 | 0.12 | 289 | -2.142 | 0.083 |
| I - N | Map_7 | Children | 0.007 | 0.121 | 289 | 0.055 | 0.998 |
| C - I | Map_8 | Children | 0.393 | 0.062 | 289 | 6.361 | <0.001 |
| C - N | Map_8 | Children | -0.181 | 0.05 | 289 | -3.589 | 0.001 |
| I - N | Map_8 | Children | -0.574 | 0.06 | 289 | -9.555 | <0.001 |
| C - I | Map_9 | Children | -0.105 | 0.057 | 289 | -1.857 | 0.153 |
| C - N | Map_9 | Children | 0.424 | 0.07 | 289 | 6.084 | <0.001 |
| I - N | Map_9 | Children | 0.53 | 0.064 | 289 | 8.219 | <0.001 |
| C - I | Map_10 | Children | -0.004 | 0.078 | 289 | -0.055 | 0.998 |
| C - N | Map_10 | Children | 0.233 | 0.083 | 289 | 2.805 | 0.015 |
| I - N | Map_10 | Children | 0.237 | 0.081 | 289 | 2.92 | 0.011 |
| C - I | Map_7 | Older Adults | -0.247 | 0.051 | 289 | -4.86 | <0.001 |
| C - N | Map_7 | Older Adults | 0.012 | 0.055 | 289 | 0.212 | 0.976 |
| I - N | Map_7 | Older Adults | 0.259 | 0.053 | 289 | 4.905 | <0.001 |
| C - I | Map_8 | Older Adults | -0.024 | 0.093 | 289 | -0.254 | 0.965 |
| C - N | Map_8 | Older Adults | 0.415 | 0.092 | 289 | 4.508 | <0.001 |
| I - N | Map_8 | Older Adults | 0.439 | 0.102 | 289 | 4.316 | <0.001 |
| C - I | Map_9 | Older Adults | -0.729 | 0.121 | 289 | -6.031 | <0.001 |
| C - N | Map_9 | Older Adults | -0.729 | 0.117 | 289 | -6.219 | <0.001 |
| I - N | Map_9 | Older Adults | 0 | 0.116 | 289 | 0.001 | 1 |
| C - I | Map_10 | Older Adults | 0.17 | 0.058 | 289 | 2.928 | 0.01 |
| C - N | Map_10 | Older Adults | -0.197 | 0.054 | 289 | -3.658 | 0.001 |
| I - N | Map_10 | Older Adults | -0.368 | 0.056 | 289 | -6.622 | <0.001 |
| C - I | Map_7 | Young Adults | -0.013 | 0.049 | 289 | -0.262 | 0.963 |
| C - N | Map_7 | Young Adults | -0.062 | 0.048 | 289 | -1.3 | 0.396 |
| I - N | Map_7 | Young Adults | -0.049 | 0.048 | 289 | -1.02 | 0.565 |
| C - I | Map_8 | Young Adults | 0.009 | 0.082 | 289 | 0.104 | 0.994 |
| C - N | Map_8 | Young Adults | 0.203 | 0.078 | 289 | 2.585 | 0.028 |
| I - N | Map_8 | Young Adults | 0.194 | 0.084 | 289 | 2.307 | 0.056 |
| C - I | Map_9 | Young Adults | 0.476 | 0.192 | 289 | 2.476 | 0.037 |
| C - N | Map_9 | Young Adults | 0.041 | 0.161 | 289 | 0.253 | 0.965 |
| I - N | Map_9 | Young Adults | -0.435 | 0.142 | 289 | -3.058 | 0.007 |
| C - I | Map_10 | Young Adults | -0.19 | 0.061 | 289 | -3.13 | 0.005 |
| C - N | Map_10 | Young Adults | 0.169 | 0.069 | 289 | 2.463 | 0.038 |
| I - N | Map_10 | Young Adults | 0.359 | 0.065 | 289 | 5.54 | <0.001 |
