# Supplementary material for: The temporal dynamics of the Stroop effect from childhood to young and older adulthood
Source: PLoS One. 2023 Mar 30;18(3):e0256003. doi: 10.1371/journal.pone.0256003 (PMC10062650; doi:10.1371/journal.pone.0256003)
Supplement: S9 Table — The R command of the model is transcribed on the first row. (DOCX) [file pone.0256003.s014.docx]

| ***Model:*** *glmer(Onset ~ conditions*age groups*maps + (1\|Subjects ID), family = Gamma(link = “log”), data = data stimulus-aligned, glmerControl(optimizer = “bobyqa”, calc.derivs = FALSE)* | | | |
| --- | --- | --- | --- |
| **Effects** | **Chisq** | **Df** | **Pr(>Chisq)** |
| Conditions | 1.844 | 2 | 0.398 |
| Age group | 10.123 | 2 | 0.006 |
| Maps | 256.062 | 5 | <0.001 |
| Conditions*age group | 7.498 | 4 | 0.112 |
| Conditions*maps | 8.243 | 10 | 0.605 |
| Age group*maps | 129.292 | 10 | <0.001 |
| Conditions*age group*maps | 11.853 | 20 | 0.921 |
