# Supplementary material for: The temporal dynamics of the Stroop effect from childhood to young and older adulthood
Source: PLoS One. 2023 Mar 30;18(3):e0256003. doi: 10.1371/journal.pone.0256003 (PMC10062650; doi:10.1371/journal.pone.0256003)
Supplement: S10 Table — (DOCX) [file pone.0256003.s015.docx]

| **Contrast** | **Maps** | **Estimate** | **SE** | **df** | **z ratio** | **p value** |
| --- | --- | --- | --- | --- | --- | --- |
| Children - Older Adults | Map1 | 0.345 | 0.067 | Inf | 5.146 | <0.001 |
| Children - Young Adults | Map1 | 0.285 | 0.072 | Inf | 3.96 | <0.001 |
| Older Adults - Young Adults | Map1 | -0.061 | 0.057 | Inf | -1.07 | 0.533 |
| Children - Older Adults | Map2 | -0.109 | 0.06 | Inf | -1.83 | 0.16 |
| Children - Young Adults | Map2 | 0.151 | 0.058 | Inf | 2.592 | 0.026 |
| Older Adults - Young Adults | Map2 | 0.26 | 0.071 | Inf | 3.686 | 0.001 |
| Children - Older Adults | Map3 | -0.076 | 0.043 | Inf | -1.798 | 0.17 |
| Children - Young Adults | Map3 | -0.234 | 0.045 | Inf | -5.238 | <0.001 |
| Older Adults - Young Adults | Map3 | -0.158 | 0.043 | Inf | -3.634 | 0.001 |
| Children - Older Adults | Map4 | 0.043 | 0.051 | Inf | 0.846 | 0.674 |
| Children - Young Adults | Map4 | 0.242 | 0.053 | Inf | 4.565 | <0.001 |
| Older Adults - Young Adults | Map4 | 0.2 | 0.056 | Inf | 3.552 | 0.001 |
| Children - Older Adults | Map5 | 0.165 | 0.071 | Inf | 2.319 | 0.053 |
| Children - Young Adults | Map5 | 0.188 | 0.065 | Inf | 2.878 | 0.011 |
| Older Adults - Young Adults | Map5 | 0.023 | 0.051 | Inf | 0.455 | 0.892 |
| Children - Older Adults | Map6 | 0.205 | 0.087 | Inf | 2.345 | 0.05 |
| Children - Young Adults | Map6 | 0.278 | 0.054 | Inf | 5.2 | <0.001 |
| Older Adults - Young Adults | Map6 | 0.073 | 0.087 | Inf | 0.844 | 0.676 |
