# Supplementary material for: The temporal dynamics of the Stroop effect from childhood to young and older adulthood
Source: PLoS One. 2023 Mar 30;18(3):e0256003. doi: 10.1371/journal.pone.0256003 (PMC10062650; doi:10.1371/journal.pone.0256003)
Supplement: S11 Table — The R command of the model is transcribed on the first row. (DOCX) [file pone.0256003.s016.docx]

| ***Model:*** *glmer(Onset ~ conditions*age groups*maps + (1\|Subjects ID), family = Gamma(link = “log”), data = data response-aligned, glmerControl(optimizer = “bobyqa”, calc.derivs = FALSE)* | | | |
| --- | --- | --- | --- |
| Effects | Chisq | Df | Pr(>Chisq) |
| Conditions | 8.29 | 2 | 0.016 |
| Age group | 3.033 | 2 | 0.219 |
| Maps | 113.837 | 3 | <0.001 |
| Conditions*age_group | 3.34 | 4 | 0.503 |
| Conditions*maps | 8.036 | 6 | 0.235 |
| Age group*maps | 33.561 | 6 | <0.001 |
| Conditions*age group*maps | 23.961 | 12 | 0.021 |
